# Protein Synthesis in *E. coli*: Dependence of Codon-Specific Elongation on tRNA Concentration and Codon Usage

**DOI:** 10.1371/journal.pone.0134994

**Published:** 2015-08-13

**Authors:** Sophia Rudorf, Reinhard Lipowsky

**Affiliations:** Theory and Bio-Systems, Max Planck Institute of Colloids and Interfaces, Potsdam, Germany; University of Lethbridge, CANADA

## Abstract

To synthesize a protein, a ribosome moves along a messenger RNA (mRNA), reads it codon by codon, and takes up the corresponding ternary complexes which consist of aminoacylated transfer RNAs (aa-tRNAs), elongation factor Tu (EF-Tu), and GTP. During this process of translation elongation, the ribosome proceeds with a codon-specific rate. Here, we present a general theoretical framework to calculate codon-specific elongation rates and error frequencies based on tRNA concentrations and codon usages. Our theory takes three important aspects of *in-vivo* translation elongation into account. First, non-cognate, near-cognate and cognate ternary complexes compete for the binding sites on the ribosomes. Second, the corresponding binding rates are determined by the concentrations of free ternary complexes, which must be distinguished from the total tRNA concentrations as measured *in vivo*. Third, for each tRNA species, the difference between total tRNA and ternary complex concentration depends on the codon usages of the corresponding cognate and near-cognate codons. Furthermore, we apply our theory to two alternative pathways for tRNA release from the ribosomal E site and show how the mechanism of tRNA release influences the concentrations of free ternary complexes and thus the codon-specific elongation rates. Using a recently introduced method to determine kinetic rates of *in-vivo* translation from *in-vitro* data, we compute elongation rates for all codons in *Escherichia coli*. We show that for some tRNA species only a few tRNA molecules are part of ternary complexes and, thus, available for the translating ribosomes. In addition, we find that codon-specific elongation rates strongly depend on the overall codon usage in the cell, which could be altered experimentally by overexpression of individual genes.

## Introduction

**Fig 1:**
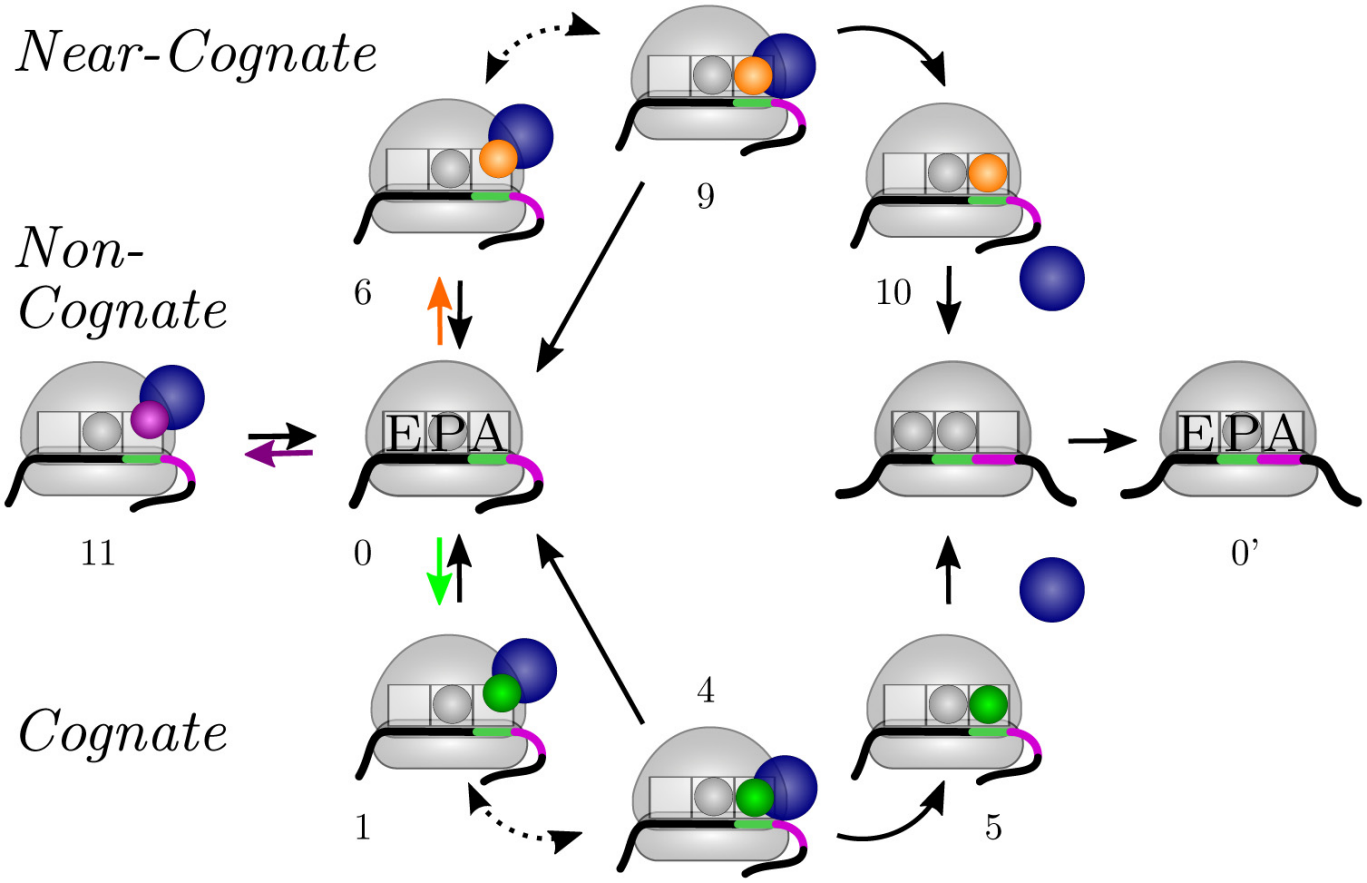
Translation elongation cycle. The ribosome has three tRNA binding sites, the A, P, and E site. A ribosome that has just arrived at a new (green) codon of an mRNA (state “0”) has an empty A site, whereas the P site is occupied by a tRNA (here shown as small gray sphere) that is cognate or near-cognate to the preceding codon. Elongation factor EF-Tu (blue spheres), aa-tRNAs (green, orange, and purple small spheres), and GTP molecules (not shown) form ternary complexes. Free cognate, near-cognate and non-cognate ternary complexes bind to the ribosome with rates depending on their respective concentrations (green, orange, and purple arrows from state “0” to states “1”, “6”, and “11”, respectively). Since the initial binding is not codon-specific, all kinds of ternary complexes unbind again from the ribosome with the same dissociation rate. Alternatively, a cognate or near-cognate ternary complex can be recognized by the ribosome (dotted arrows from states “1” and “6” to states “4” and “9”, respectively) before the ternary complex is either completely released (arrows from states “4” and “9” to state “0”), brought back to the initial binding state (dotted arrows from states “4” and “9’ to states “1” and “6”, respectively), or its aa-tRNA is accommodated in the ribosomal A site (arrows from states “4” and ”9” to states “5” and “10”, respectively). Along with aa-tRNA accommodation, EF-Tu leaves the ribosome. The new A-site tRNA is then further processed and shifted to the P site, while the ribosome translocates to the next (purple) codon. The former P-site tRNA is now in the E site. Depending on the assumed pathway of tRNA release, the E-site tRNA either dissociates very rapidly from the ribosome (2-1-2 pathway), or stays until the next aa-tRNA has been accommodated in the ribosomal A site (2-3-2 pathway). The numerals correspond to the ribosomal states of the codon-specific Markov process introduced below.

In the past decades, the function and structure of the ribosome were intensively studied [1, 2]. Each ribosome consists of a small and a large subunit, both of which contain RNA and protein molecules. During the initiation of translation, the two subunits bind to the mRNA upstream of its coding sequence and then form a functional ribosome at the start codon of the mRNA [3]. The subsequent codon-wise movement of the ribosome along the mRNA is called translation elongation. Many *in-vitro* experiments have been performed to identify the individual steps of the elongation process and to reveal the corresponding kinetics [4–7]. Our current view of this process is summarized in Fig 1.

**Fig 2:**
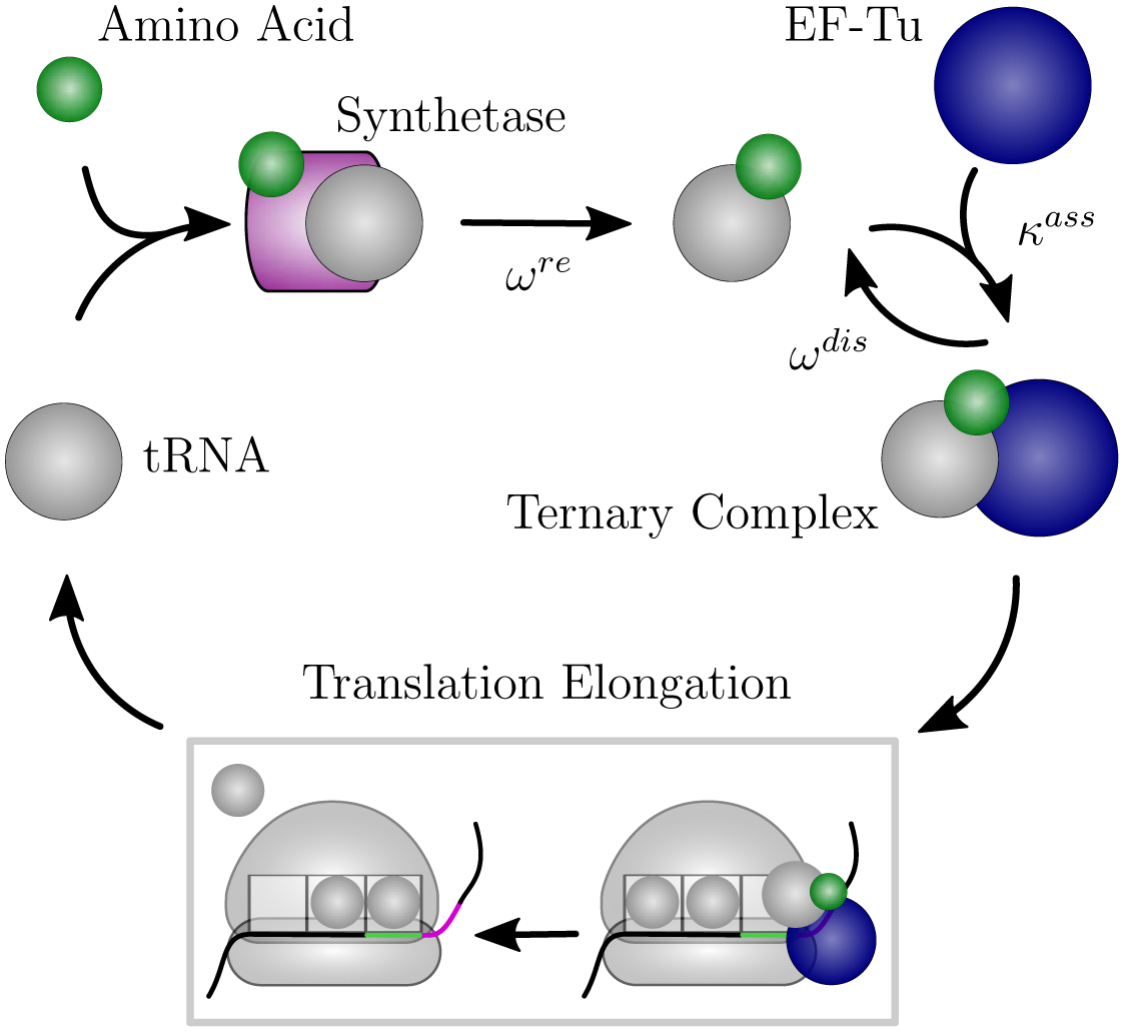
Recharging cycle of tRNAs. During translation elongation an aa-tRNA delivers its amino acid to the elongating peptide chain. After the processed tRNA is released from a ribosomal E site, it binds to an aminoacyl tRNA synthetase. The synthetase recharges the tRNA with a new amino acid, and the recharged aa-tRNA binds to elongation factor EF-Tu to form a new ternary complex.

First, a ternary complex consisting of elongation factor EF-Tu, an aminoacyl tRNA (aa-tRNA), and a GTP molecule binds to the ribosome. If the aa-tRNA is cognate and matches the currently read codon, it its accommodated in the ribosomal A site via GTP hydrolysis. The peptide chain is then extended by forming a peptide bond between the amino acid of the “old” tRNA in the ribosome’s P site and the amino acid of the “new” tRNA in the A site. Another elongation factor (EF-G) binds to the ribosome and, fueled by a second GTP hydolysis, causes it to translocate to the next codon. Simultaneously, the A-site tRNA is pushed to the P site and the P-site tRNA is moved to the E site (exit), from where it leaves the ribosome. The ribosomal A and P sites are related to the core tasks of a ribosome, i.e., decoding and peptide bond formation. The function of the E site is not yet fully understood but it is believed that it supports translocation of the ribosome [2]. It is widely assumed that the E-site tRNA is released from the ribosome before a new ternary complex binds to the A site. This was named *2-1-2* pathway of E-site tRNA release and has been corroborated by several *in-vitro* experiments [8–11]. However, for the early cycles of elongation corresponding to a relatively short length of the nascent peptide chain, the alternative *2-3-2* pathway has also been observed [10]. In this pathway, the binding of a new ternary complex to the A site precedes the release of the E-site tRNA [12]. After a tRNA is released from the ribosomal E site, it is recharged with an amino acid by an aminoacyl tRNA synthetase and forms a new ternary complex by binding to an EF-Tu molecule, see Fig 2.

Translation is terminated as soon as the ribosome encounters one of the three stop codons. Release factors unbind the peptide chain from the last P-site tRNA and the two ribosome subunits dissociate from the mRNA [13].

Protein synthesis must comply with high requirements concerning speed and accuracy. It must be fast enough to ensure doubling of protein mass within the time scale of cell division. At the same time, the synthesis of proteins must be very precise, because erroneous proteins are often dysfunctional or even harmful to the cell. Therefore, perturbations that hamper or dysregulate protein synthesis can lead to all kinds of cellular defects up to cell death. Indeed, a well-known strategy to fight bacterial infections is based on antibiotics that block protein synthesis in bacteria, hence killing the microorganisms. On the other hand, many diseases in multicellular organisms arise from genetic mutations that affect protein synthesis. Thus, it is of great general interest to understand what determines the speed and accuracy of protein synthesis.

Although each elongation cycle involves basically the same steps, the speed of translation is not uniform. For two decades, it has been known that *in-vivo* elongation rates are codon-specific, i.e., the local velocity of the ribosome depends on the codon that is translated [14, 15]. Many mechanisms have been proposed to explain this variability in the speed of translation, of which a recent review can be found in [16, 17]. For example, mRNA is often folded into a secondary structure which the ribosome has to open up before it can translate the message [18, 19]. Furthermore, certain codon sequences may interact with the ribosome and slow it down [20]. Also, the peptide chain can interact with the ribosome, especially the chain segment that is still in the ribosomal exit tunnel [21]. In addition, ribosomes can be slowed down by preceding ribosomes translating the same message if they come into close contact [22]. Equivalently, codon- or tRNA-dependent variations in the ribosomal processing, and the presence of co-translational processes, like chaperone interactions or co-transcriptional translation, could affect the efficiency of the elongation process [23, 24].

One additional factor is believed to determine the local speed of translation, namely the concentration of ternary complexes. In contrast to all of the other influencing factors mentioned above, the concentration of ternary complexes represents a universal control of the speed of translation, independent of the mRNA that is translated or the organism that is studied. Because the ribosome has to wait for a cognate ternary complex to bind before it can proceed, it is plausible that translation is faster when more ternary complexes are present. Based on this idea the tRNA adaptation index (tAI) was introduced and interpreted as a measure for the actual codon-specific elongation rate [25, 26].

From a theoretical point of view, not only the cognate, but also the non- and near-cognate ternary complexes should have an effect on the elongation rate of the corresponding codon, as they can bind to the ribosome as well. Experimentally, their influence has still to be clarified [27–29]. The competition between cognate, near- and non-cognate aa-tRNAs was studied theoretically and was found to strongly affect codon-specific elongation rates [30, 31].

In addition to the effect of near- and non-cognate aa-tRNAs, the competition between the active ribosomes for ternary complexes also has a strong influence on the elongation speed. Each *Escherichia coli* (*E. coli*) cell contains about twenty to thirty thousand ribosomes actively translating at the same time. As they always keep one tRNA in their P site and sometimes a tRNA in their E site, active ribosomes dramatically reduce the concentrations of tRNAs that can form ternary complexes. Therefore, the simultaneous action of ribosomes can turn abundant into rare ternary complexes. Thus, elongation rates should rather depend on the concentrations of available ternary complexes than on the total amount of tRNAs.

Here, we develop a theoretical framework that allows us to determine the codon-specific speed of translation based on the total, experimentally measured tRNA concentrations. Previously published theories [30–35] on tRNA concentration dependent elongation rates have some limitations because they ignored fundamental aspects of ribosome translation. These aspects include (i) the proper distinction between the concentration of free ternary complexes and the measured tRNA concentrations, a distinction that was not considered in [30, 33]; (ii) the dependence of the free ternary complexes on the recharging of deacetylated tRNA by new amino acids which was not taken into account in [30–32]; (iii) the different *in-vitro* and *in-vivo* values for the rates of aa-tRNA decoding, accommodation, peptide bond formation and translocation [36], a difference that was ignored in [30–32]; and (iv) the competition between cognate, near-, and non-cognate ternary complexes for initial binding to ribosomes, which has a strong influence on the translation process [31] but was ignored in [32, 34, 35]. In the present paper, we include all of these aspects within a single theoretical framework.

In this paper, we will develop a comprehensive theory on translation elongation that takes into account all of the aforementioned mechanisms that influence the concentrations of ternary complexes, as well as the competition of ternary complexes. Furthermore, we use kinetic rates to describe *in-vivo* translation that were deduced from their corresponding measured *in-vitro* rates by applying a recently published extremum principle, i.e., by minimizing the kinetic distance of the *in-vitro* and *in-vivo* rates [36]. Our theory sheds light on the intricate relationships between the internal dynamics of the ribosome, the availability of ternary complexes, and the codon usages, both for the 2-1-2 and for the 2-3-2 pathway of tRNA release from the E site.

## Results

### Codons and tRNAs: Some Definitions

We denote by **C** the set of sense codons which are labeled by *c* = 1, 2, … |**C**|, where in *E. coli* |**C**| = 61. The different species of tRNA form the set **A**. These tRNA species will be distinguished by the label *a* = 1, 2, … |**A**|. The total number |**A**| of different tRNA species depends on the organism. In *E. coli*, there are |**A**| = 43 different elongator tRNA molecules [37]. For each codon *c*, there is a set **A**_co_ (*c*) of cognate tRNAs. All other tRNAs belong either to the set **A**_nr_ (*c*) of near-cognate or to the set **A**_no_ (*c*) of non-cognate tRNAs. Therefore, each codon *c* ∈ **C** leads to a unique partition of the set **A** into pairwise disjoint sets {**A**_co_ (*c*), **A**_nr_ (*c*), **A**_no_ (*c*)}.

**Fig 3:**
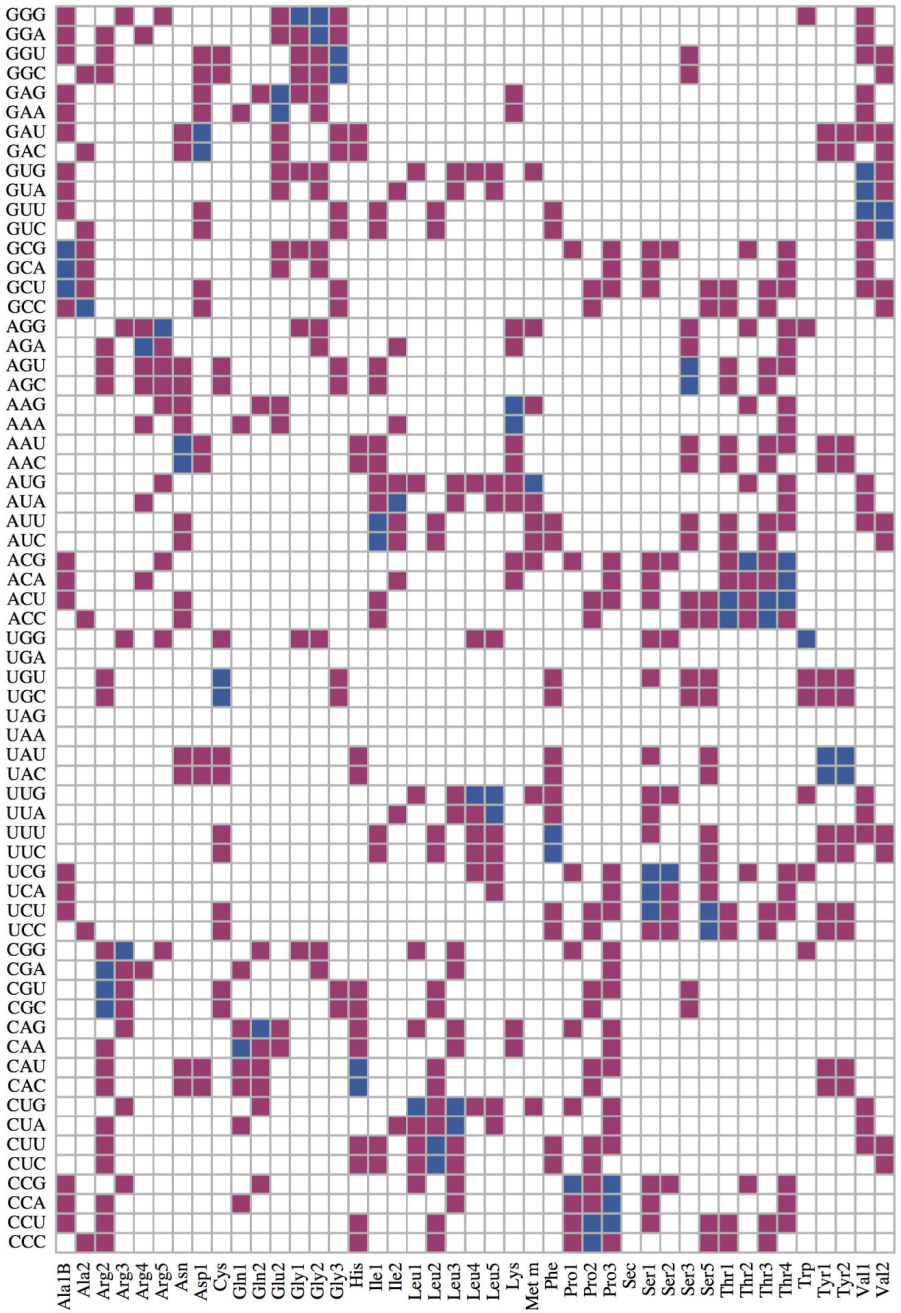
Cognate (blue), near-cognate (purple), and non-cognate tRNAs (white). for all sense codons in *E. coli* following the definitions of “cognate” and “near-cognate” as given in [37] and [38], respectively.

In some cases, a certain tRNA species is cognate to more than one codon. The set of codons that are cognate to the tRNA with index *a* is denoted by **C**_co_ (*a*), whereas the near- and non-cognate codons are contained in the sets **C**_nr_ (*a*) and **C**_no_ (*a*), respectively. Therefore, each tRNA *a* ∈ **A** leads to a unique partition of the set **C** into pairwise disjoint sets {**C**_co_ (*a*), **C**_nr_ (*a*), **C**_no_ (*a*)}. The complex relationships between cognate, near- and non-cognate tRNAs and codons can be visualized by a large matrix as displayed in Fig 3 for *E. coli*. Note that this matrix has 43 × 61 = 2623 elements.

### Theoretical Description of the Elongation Cycle

**Fig 4:**
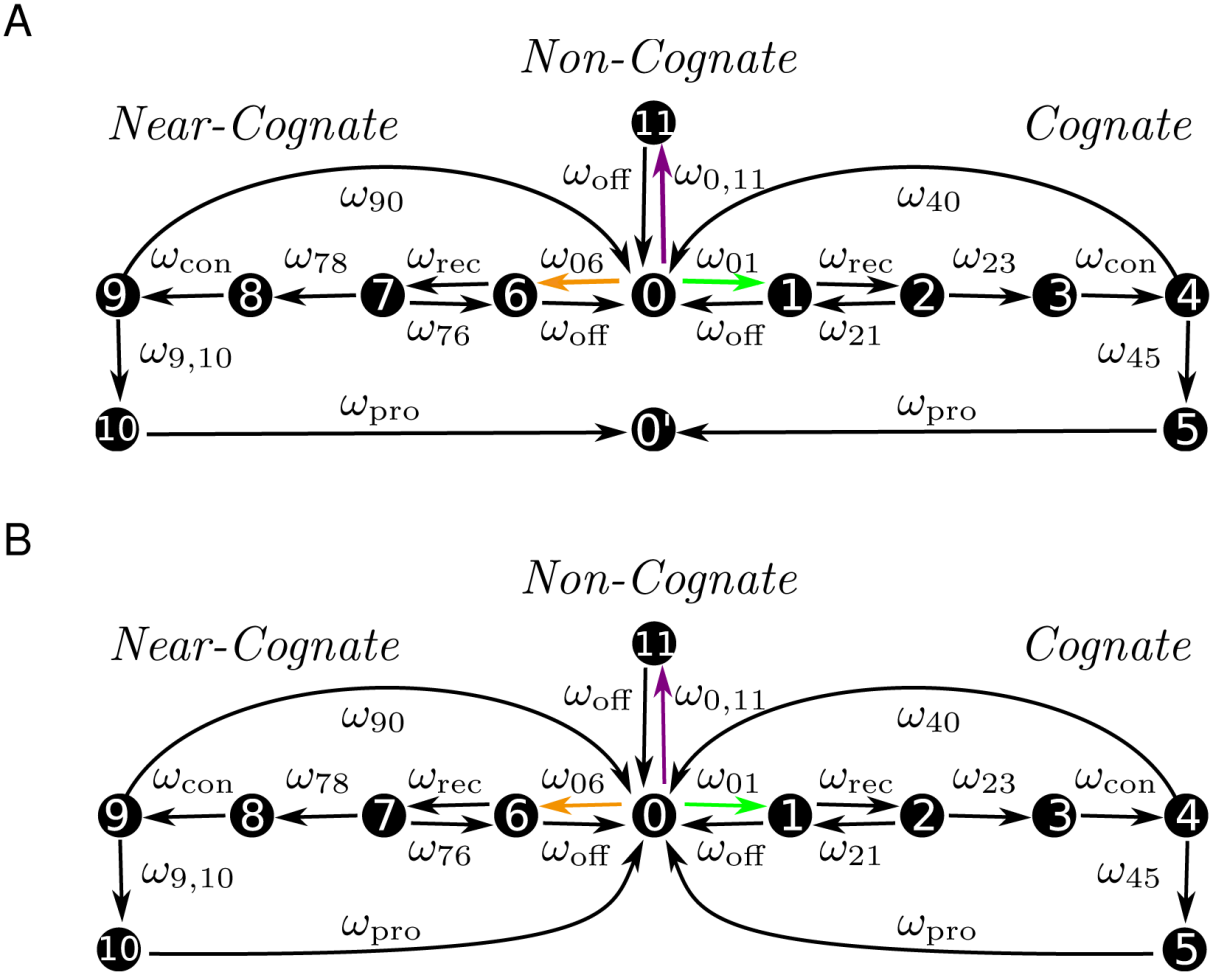
Representation of translation elongation as a Markov process. (A) Each of the substeps of the elongation cycle is represented by one state in the Markov model, which leads to twelve states per sense codon. State (*c*|0) indicates the state assumed by a ribosome reading codon *c* when it is not bound to a ternary complex. State (*c*′|0′) is attained by a ribosome after translocation to the next codon *c*′. All rates of transitions between states are taken to be codon-independent, except for the binding rates of cognate, near-, and non-cognate ternary complexes (green, orange and purple arrows). (B) Auxiliary Markov process for the computation of dwell times. The auxiliary Markov process is almost identical to the Markov process of codon-specific elongation depicted in (A), but the absorbing state (*c*′|0′) is omitted. The states (*c*|5) and (*c*|10) are directly connected back to the initial state (*c*|0) with the same transition rate ω_pro_ as in the original process.

We model translation as a continuous-time Markov process as introduced in [36] to capture the stochastic nature of the ribosomal movement. In contrast to earlier stochastic theories of translation [39, 40], our model of translation elongation as depicted in Fig 1 is based on the detailed chemical kinetics as determined for a certain *in-vitro* assay [7, 41–46]. For each sense codon *c*, the ribosome can cycle through twelve ribosomal states, numbered from 0 to 11 as shown in Fig 4(A). Each state of the translating ribosome is characterized by the codon in its A site and by the binding of ternary complexes, aa-tRNAs, or tRNAs to the ribosomal A, P, and E sites. A ribosome that has just moved to a codon *c* has a free A site, but its P site is occupied by a tRNA that is cognate or near-cognate to the preceding codon. The corresponding ribosomal state is denoted by (*c*|0).

When a ternary complex binds to the ribosome, it can be cognate, near-cognate, or non-cognate to the codon in the ribosomal A site. After binding a cognate ternary complex, the ribosome arrives in state (*c*|1); the binding of a near-cognate ternary complex leads to state (*c*|6); finally, if the bound ternary complex is non-cognate to *c*, the ribosome is in state (*c*|11). A non-cognate ternary complex always dissociates from the ribosome which then goes back to state (*c*|0). After initial binding, a cognate ternary complex has to be recognized by the ribosome, which is achieved in state (*c*|2). The recognition is followed by an activation of GTPase and GTP hydrolysis, leading to ribosomal state (*c*|3), and release of phosphate and rearrangements of the EF-Tu molecule during the transition from (*c*|3) to state (*c*|4). From here, the aa-tRNA gets accommodated and the EF-Tu is released with high probability, leading to state (*c*|5). However, in rare cases, the ternary complex is released from the ribosome which then returns to state (*c*|0). We will call the states (*c*|1) to (*c*|5) the cognate branch of the Markov process. In addition to the cognate branch, the Markov process also involves an analogous near-cognate branch of another five states: a near-cognate ternary complex is recognized in state (*c*|7) and gets usually rejected during this initial selection step, bringing the ribosome back to state (*c*|0). Alternatively, GTPase is activated, GTP is hydrolyzed and the ribosome with the near-cognate ternary complex proceeds to state (*c*|8). After release of phosphate and EF-Tu rearrangements during the transition to state (*c*|9), the ribosome usually rejects the near-cognate ternary complex and returns to state (*c*|0). The latter step is called proofreading. If the ribosome with the near-cognate ternary complex moves on to state (*c*|10), the near-cognate aa-tRNA becomes fully accommodated in the ribosomal A site and the EF-Tu leaves the ribosome, resulting in a misreading error. After cognate or near-cognate aa-tRNA accommodation, the A-site tRNA is processed which includes peptide bond formation and translocation of the ribosome to the next codon along with a transfer of the A-site tRNA to the P site and of the P-site tRNA to the E site. A new elongation cycle on the following codon *c*′ can begin, indicated by the ribosomal state (*c*′|0′) in Fig 4(A). Processed tRNAs leave the ribosome from the E site. Afterwards, they bind to synthetases that recharge them with amino acids, before the recharged aa-tRNAs bind again to EF-Tu to form new ternary complexes, see Fig 2.

Note that some transitions from one state of the Markov process to another state are reversible, which is depicted in Fig 4(A) by pairs of opposing arrows. In contrast, several other transitions are taken to be irreversible as indicated by single arrows. These assumptions of irreversibility are based on experimental findings, i.e., these transitions are irreversible in the sense that the rates of the reverse transitions are too small to be resolved experimentally.

We restrict our theoretical analysis to elongation under low densities of translating ribosomes on mRNAs, i.e., elongation without ribosome-ribosome interactions. In fact, for eukaryotic translation experimental findings suggest that most ribosomes do not interfere under physiological conditions [47]. However, interactions of ribosomes can be easily taken into account if the Markov process is studied by stochastic simulations as for example described in [36, 40, 48]. In [36], we used such simulations to study the time-dependent incorporation of radioactively labeled amino acids into proteins.

### Binding Rates and Internal Transition Rates

The ribosomal transition rate from state *i* to state *j* will be denoted by *ω*_ij_. Thus, the rates *ω*_01_, *ω*_06_, and *ω*_0,11_ govern the transition from (*c*|0) to (*c*|1), (*c*|6) and (*c*|11), respectively. The rates *ω*_01_, *ω*_06_, and *ω*_0,11_ depend on the molar concentrations ${\hat{\text{X}}}_{a}$ of available, free ternary complexes containing aa-tRNA species *a* and have the form

$$\omega_{01}=\;\kappa_{\text{on}}\sum_{a\in A_{\text{co}}\left(c\right)}{\hat{X}}_{a},$$ (1)

$$\omega_{06}=\;\kappa_{\text{on}}\sum_{a\in A_{\text{nr}}\left(c\right)}{\hat{X}}_{a},$$ (2)

and

$$\omega_{0,11}=\;\kappa_{\text{on}}\sum_{a\in A_{\text{no}}\left(c\right)}{\hat{X}}_{a},$$ (3)

where *κ*_on_ is the binding rate constant that we take to be identical for all (cognate, near-cognate, and non-cognate) ternary complexes in agreement with experimental observations [49]. The sets **A**_co_ (*c*), **A**_nr_ (*c*), and **A**_no_ (*c*) contain the tRNA species that are cognate, near-cognate, and non-cognate to codon *c* as previously defined.

In general, transition rates in the cognate branch of the Markov process differ from their counterparts in the near-cognate branch. As an exception to this rule, based on experimental findings [41, 44] we take the transition rates *ω*_12_ and *ω*_67_ to state (*c*|2) and (*c*|7) and the transition rates *ω*_34_ and *ω*_89_ to state (*c*|4) and (*c*|9) to be identical for all cognate and near-cognate ternary complexes. The same assumption is made for the transitions from the states (*c*|5) and (*c*|10) to the free state (*c*′|0′) after translocation

$$\omega_{12}=\omega_{67}\equiv \omega_{\text{rec}},$$ (4)

$$\omega_{34}=\omega_{89}\equiv \omega_{\text{con}},$$ (5)

and

$$\omega_{50\prime }=\omega_{10,0\prime }\equiv \omega_{\text{pro}}.$$ (6)

Furthermore, the dissociation rates from state (*c*|1), (*c*|6), or (*c*|11) back to state (*c*|0) are taken to be identical for all cognate, near-cognate, and non-cognate ternary complexes [44]

$$\omega_{10}=\omega_{60}=\omega_{11,0}\equiv \omega_{\text{off}},$$ (7)

because dissociation happens before a physical contact between the A-site codon and the anti-codon of the aa-tRNA is made and, thus, before a distinction between cognate, near-cognate, and non-cognate ternary complexes is possible. In summary, the 20 transitions of the Markov process in Fig 4(A) are governed by three codon-dependent binding rates *ω*_01_, *ω*_06_, and *ω*_0,11_, and 12 codon-independent internal rates. Numerical values for the latter 12 internal rates are given in S1 Table in the Supporting Information. They were obtained by minimization of the kinetic distance as introduced and described in [36].

### Codon-Specific Elongation Rates and Error Frequencies

We define the elongation time t_*c*,elo_ of codon *c* as the average time that is needed to finish a complete elongation cycle on codon *c*. According to the Markov process described in the previous sections, this time is the average time to absorption in state (*c*′|0′) from state (*c*|0). We define the codon-specific elongation rate *ω*_*c*,elo_ as the inverse of the elongation time t_*c*,elo_. The codon-specific elongation time can be computed as a mean first passage time using the general theory of Markov processes with absorption [50, 51]. Numerical results for the the codon-specific elongation rates *ω*_*c*,elo_ are given in S2 and S3 Tables in the Supporting Information. Alternatively, we can express the elongation time by the sum of the average dwell times t_(*c*|*i*)_ in states (*c*|*i*) with *i* = 0, 1, …, 11 per elongation cycle on codon *c*. This leads to

$$\omega_{c,\text{elo}}\equiv {\text{t}}_{c,\text{elo}}^{-1}\equiv {\left(\overset{11}{\underset{i=0}{\sum }}{\text{t}}_{\left(c|i\right)}\right)}^{-1}.$$ (8)

To compute the dwell times t_(*c*|*i*)_, we introduce an auxiliary Markov process and study its steady state as described by Hill [52]. The auxiliary Markov process is almost identical to the process depicted in Fig 4(A) with one modification: In the auxiliary process, the absorbing state (*c*′|0′) is identified with the state (*c*|0), see Fig 4(B). Thus, when the ribosome arrives on state (*c*′|0′), it is immediately transferred back to state (*c*|0). Instead of the transitions (*c*|5) to (*c*′|0′) and (*c*|10) to (*c*′|0′), the states (*c*|5) and (*c*|10) are connected back to the initial state (*c*|0) using the same rate *ω*_pro_ as in the original process. In this way, the states (*c*|*i*) are decoupled from all other states that the ribosome visited prior to codon *c* under consideration. Such a decoupling is possible because the Markov process has no memory. The time dependence of the probabilities ${\tilde{\text{P}}}_{c,i}(t)$ to attain the states (*c*|*i*) in the auxiliary process are described by the loss-and-gain (or master) equations

$$\frac{d}{dt}\;{\tilde{P}}_{c,i}\left(t\right)=\sum_{j}\;({\tilde{P}}_{c,j}\left(t\right)\;\omega_{\text{ji}}-{\tilde{P}}_{c,i}\left(t\right)\;\omega_{\text{ij}}),$$ (9)

where the transition rates *ω*_ij_ are defined in the previous section and Fig 4(B). Furthermore, the time dependent probabilities ${\tilde{\text{P}}}_{c,i}(t)$ fulfill the normalization condition

$$\sum_{i}{\tilde{P}}_{c,i}\left(t\right)=1.$$ (10)

The dwell times t_(*c*|*i*)_ can be calculated from the steady state of the auxiliary Markov process. In the steady state, the left side of Eq (9) vanishes and this set of equations together with Eq (10) can be solved for ${\tilde{\text{P}}}_{c,0}^{\text{st}},\;{\tilde{\text{P}}}_{c,1}^{\text{st}},\;\ldots ,\;{\tilde{\text{P}}}_{c,11}^{\text{st}}$, where ${\tilde{\text{P}}}_{c,i}^{\text{st}}$ denotes the steady state value ${\tilde{\text{P}}}_{c,i}^{\text{st}}\equiv {\tilde{\text{P}}}_{c,i}(t=\infty )$. The steady state probabilities ${\tilde{\text{P}}}_{c,i}^{\text{st}}$ of the auxiliary process are identical to the fractions of time that the original process spends on average in the states (*c*|*i*) with initial state (*c*|0) and absorbing state (*c*′|0′) which implies the relation

$${\tilde{\text{P}}}_{c,i}^{\text{st}}=\frac{{\text{t}}_{\left(c|i\right)}}{{\text{t}}_{c,\text{elo}}}$$ (11)

with the codon-specific elongation time t_*c*,elo_ defined in Eq (8). Explicit expressions for the resulting dwell times are given in Eqs (S2) to (S13) in the Supporting Information.

It is convenient to express the dwell times in terms of the effective cognate and near-cognate accommodation rates defined by

$$\omega_{c,\text{co}}\equiv \omega_{01}\;\frac{\pi_{12}\pi_{23}\pi_{45}}{1-\pi_{12}\pi_{21}},$$ (12)

$$\omega_{c,\text{nr}}\equiv \omega_{06}\;\frac{\pi_{67}\pi_{78}\pi_{9,10}}{1-\pi_{67}\pi_{76}}.$$ (13)

with the probability *π*_*ij*_ of transition from state (*c*|*i*) to state (*c*|*j*) as given by

$$\pi_{ij}=\frac{\omega_{ij}}{\sum_{k}\omega_{ik}}.$$ (14)

Note that the effective accommodation rates *ω*_*c*,co_ and *ω*_*c*,nr_ are proportional to the cognate or near-cognate binding rates *ω*_01_ and *ω*_06_ multiplied by the respective probabilities that the aa-tRNA of a ternary complex that has bound to a ribosome successfully accommodates in the A site instead of dissociating from the ribosome before accommodation has happened. These probabilities can be computed by first step analysis [50].

Furthermore, we define the probabilities 𝓟_*c*,co_ and 𝓟_*c*,nr_ for cognate and near-cognate accommodation

$$P_{c,\text{co}}\equiv \frac{\omega_{c,\text{co}}}{\omega_{c,\text{co}}+\omega_{c,\text{nr}}},$$ (15)

$$P_{c,\text{nr}}\equiv \frac{\omega_{c,\text{nr}}}{\omega_{c,\text{co}}+\omega_{c,\text{nr}}}.$$ (16)

The probabilities of cognate and near-cognate accommodation 𝓟_*c*,co_ and 𝓟_*c*,nr_ fulfill the normalization condition 𝓟_*c*,co_ + 𝓟_*c*,nr_ = 1. The accommodation probability 𝓟_*c*,co_ is a measure of the codon-specific fidelity: if 𝓟_*c*,co_ = 1, the ribosome translates codon *c* without any errors and, thus, with maximal fidelity. Likewise, 𝓟_*c*,nr_ represents the codon-specific infidelity, which is also named near-cognate missense error frequency.

### Codon Usages and Overall Elongation Rate

One quantity that has been determined experimentally for *E. coli* under various growth conditions is the average elongation time per codon, 〈t_*c*,elo_〉, [53] which represents the codon-specific elongation times t_*c*,elo_ averaged over all codons in the “coding transcriptome”, i.e., the population of all mRNA molecules in the cell. This average involves the codon usages *p*_*c*_, which represent the normalized frequencies or probabilities that a randomly chosen sense codon in the mRNA population is equal to *c*. The quantities *p*_*c*_ satisfy the relations

$$0\leq p_{c}\leq 1\;\text{and}\;\sum_{c=1}^{61}p_{c}=1.$$ (17)

Numerical values of the codon usages in *E. coli* for different specific growth rates are displayed in S7 Table in the Supporting Information. The average elongation time per codon 〈t_*c*,elo_〉 and its inverse, the overall elongation rate *ω*_elo_, are then given by

$$\omega_{\text{elo}}\equiv {\langle {\text{t}}_{c,\text{elo}}\rangle }^{-1}={\left(\overset{61}{\underset{c=1}{\sum }}p_{c}{\text{t}}_{c,\text{elo}}\right)}^{-1}.$$ (18)

### Probabilities to Attain Ribosomal States

**Fig 5:**
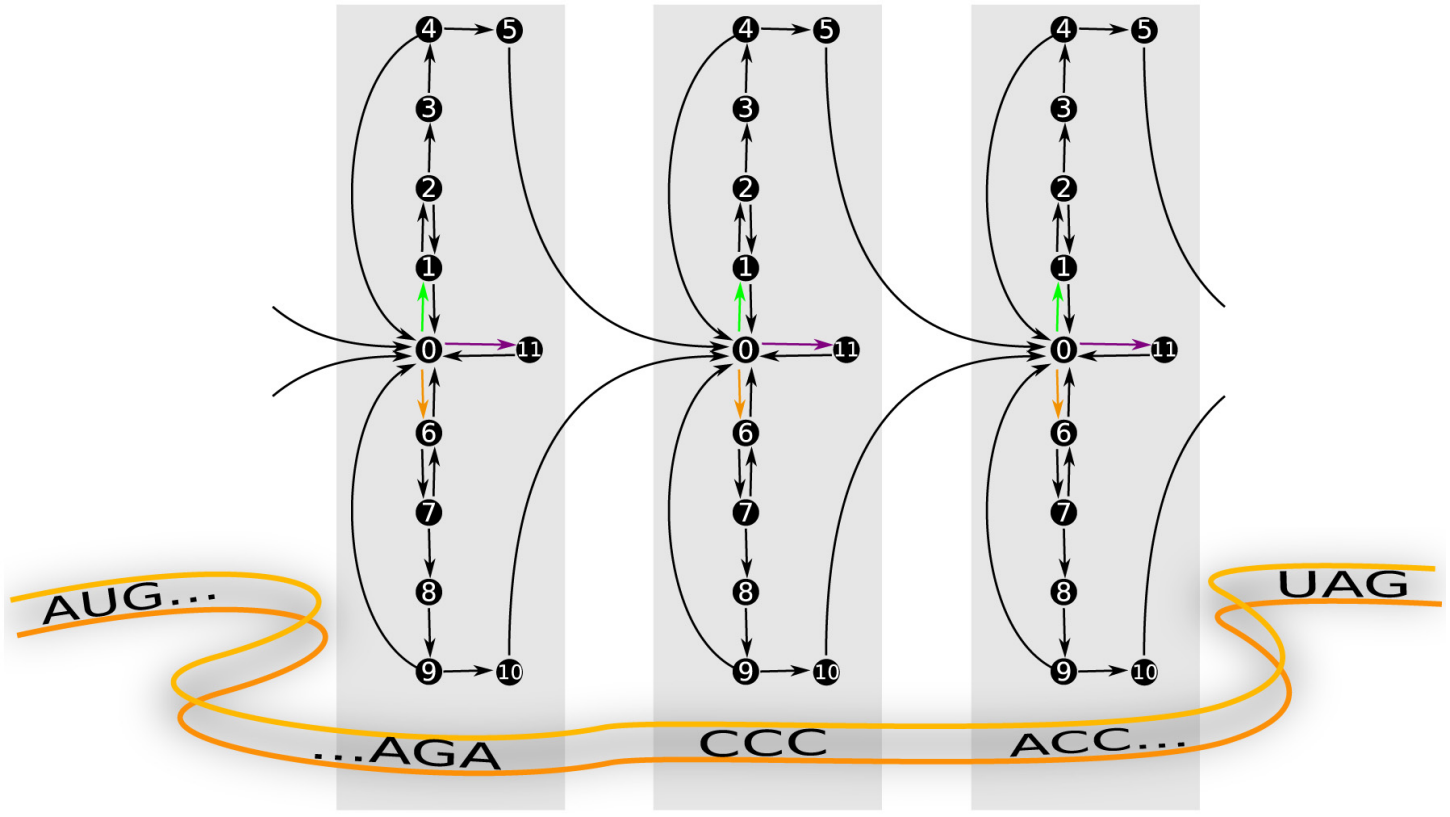
Markov process for the translation of an individual mRNA. The mRNA (orange band) consists of a sequence of codons, where in this example AUG and UAG indicate the start and the stop codon, respectively. The codons are translated sequentially from start to stop. Hence, the Markov process for the translation of an mRNA is given by the concatenation of individual codon-specific Markov processes as defined in the text and Fig 4(A).

In a cell or in an *in-vitro* translation assay, many mRNAs are usually present at the same time. The total number of all mRNA molecules will be denoted by *M*. In general, these mRNAs are composed of different codon sequences, although some of them might have the same sequence of codons and, thus, encode the same protein. To identify the state space for translation by non-interacting ribosomes, we first consider the translation of an individual mRNA *m* of length *L*_*m*_ that is composed of a sequence of codons *c*_1_, *c*_2_, *c*_3_,…*c*_*L*_*m*__, see Fig 5. For the translation process of this mRNA, the ribosomal states described above form the state space

$$S_{m}\equiv \underset{x,i}{\cup }\left\{\left(c_{x}|i\right)\right\}$$ (19)

where 1 ≤ *x* ≤ *L*_*m*_ refers to the position of the codon *c*_*x*_ ∈ **C** and *i* = 0, 1, …, 11 indicates the internal state of the ribosome as introduced above. Thus, the state space **S**_*m*_ consists of 12 × *L*_*m*_ states.

For each state (*c*_*x*_|*i*) ∈ **S**_*m*_, we can define the time-dependent probability P_*c*_*x*_,*i*_(*t*) that a ribosome dwells on codon *c*_*x*_ and attains the ribosomal state *i* at time *t*. For the Markov process described above and in Fig 5, the time dependent probabilities fulfill the following master equations

$$\frac{d}{dt}\;P_{c_{x,0}}\left(t\right)=\sum_{j=1,6,11}(P_{c_{x},j}\left(t\right)\;\omega_{\text{off}}-P_{c_{x},0}\left(t\right)\;\omega_{0j})+\sum_{j=5,10}P_{c_{x-1},j}\left(t\right)\;\omega_{\text{pro}},$$ (20)

for the free states (*c_x_*|0) and *x* ≥ 2, and

$$\frac{d}{dt}\;P_{c_{x},i}\left(t\right)=\sum_{j}(P_{c_{x},j}\left(t\right)\;\omega_{\text{ji}}-P_{c_{x},i}\left(t\right)\;\omega_{\text{ij}})\;$$ (21)

for all other states, where the transition rates *ω*_ij_ are defined in the previous section and Fig 4(A). Furthermore, the time dependent probabilities P_*c*_*x*_,*i*_(*t*) fulfill the normalization condition

$$\sum_{x,i}P_{c_{x},i}\left(t\right)=1.$$ (22)

As long as ribosome-ribosome interactions can be ignored, a full Markov model describing translation elongation on all mRNAs in the entire cell or in an *in-vitro* translation assay has a ribosomal state space **S** that is composed of all mRNA-specific state spaces **S**_*m*_

$$S\equiv \underset{m}{\cup }S_{m}=\underset{m}{\cup }\underset{x,i}{\cup }\left\{\left(c_{x,m}|i\right)\right\}$$ (23)

where the index *m* runs over all individual mRNAs 1, …, *M* and *c*_*x*,*m*_ ∈ **C** indicates the codon found at position *x* in mRNA *m*. Therefore, the full ribosomal state space of translation elongation consists of $12\times \Sigma_{m=1}^{M}L_{m}$ states, where *L*_*m*_ is the length of mRNA *m*. The average length of mRNAs in bacteria is about 300 codons [54] and there are more than 1000 mRNA molecules in each *E. coli* cell [55]. Thus, for translation in *E. coli* the state space **S** contains more than 3.6 × 10^6^ states that could be attained by each ribosome. Because of this enormous number of states, a detailed time-dependent analysis of translation elongation in the entire cell is hardly feasible.

However, we can analyze the entire Markov process of cell-wide translation elongation under steady state conditions. For a steady state, the flux of ribosomes entering the mRNAs during translation initiation is equal to the flux of ribosomes terminating translation and to the flux of ribosomes elongating on mRNAs. In particular, for every codon in the cell, the flux of arriving ribosomes balances the flux of leaving ribosomes, and this flux is the same on every codon. This means that the steady state probabilities to attain a certain state only depend on the species *c* of a codon, but not on its position *x*. In other words, all codons belonging to the same species *c* can be treated identically. Thus, in a steady state, we can describe translation by a reduced ribosomal state space that consists of 12 × 61 = 732 states (*c*|*i*), each of which corresponds to a certain codon species *c* in the ribosomal A site and one of the twelve ribosomal states *i* = 0, …, 11.

Furthermore, in a steady state, the marginal probability ${\text{P}}_{c}^{\text{st}}$ to find a ribosome on codon *c* is simply given by the relative codon-specific elongation time

$$P_{c}^{\text{st}}=\frac{p_{c}\;t_{c,\text{elo}}}{\left\langle t_{c,\text{elo}}\right\rangle },$$ (24)

with the codon-specific elongation time t_*c*,elo_, the codon usage *p*_*c*_, and the average elongation time 〈t_*c*,elo_〉, as given by the relations Eqs (8), (17), and (18), respectively. Likewise, in a steady state, the joint probability ${\text{P}}_{c,i}^{\text{st}}$ to find a ribosome on codon *c* in state (*c*|*i*) is equal to

$$P_{c,i}^{\text{st}}=P_{i|c}^{\text{st}}\;P_{c}^{\text{st}},$$ (25)

where ${\text{P}}_{i|c}^{\text{st}}$ is the conditional probability to find a ribosome in state (*c*|*i*) under the condition that codon *c* is in the ribosomal A site. The steady state conditional probabilities ${\text{P}}_{i|c}^{\text{st}}$ can be computed by using again the auxiliary Markov process introduced above and in Fig 4(B). In particular, we can identify the steady state probabilities ${\tilde{\text{P}}}_{c,i}^{\text{st}}$ of the auxiliary process with the steady state conditional probabilities ${\text{P}}_{i|c}^{\text{st}}$

$${\tilde{P}}_{c,i}^{\text{st}}=P_{i|c}^{\text{st}}.$$ (26)

Thus, in a steady state, the joint probability ${\text{P}}_{c,i}^{\text{st}}$ to find a ribosome in state (*c*|*i*) on codon *c* can be expressed by the dwell times as in Eq (11), and by the marginal probability in Eq (24), which leads to

$${\text{P}}_{c,i}^{\text{st}}=\frac{{\text{t}}_{\left(c|i\right)}}{{\text{t}}_{c,\text{elo}}}\frac{p_{c}{\text{t}}_{c,\text{elo}}}{\langle {\text{t}}_{c,\text{elo}}\rangle }=\frac{p_{c}{\text{t}}_{\left(c|i\right)}}{\langle {\text{t}}_{c,\text{elo}}\rangle }.$$ (27)

### Steady State Concentrations of Free Ternary Complexes

It is important to note that the codon-specific elongation rates defined in Eq (8) and, thus, the expression for the overall elongation rate *ω*_elo_ as in Eq (18) involve the binding rates *ω*_01_, *ω*_06_, and *ω*_0,11_ for cognate, near-cognate, and non-cognate ternary complexes. The latter rates are given by expressions Eqs (1)–(3), which depend on the concentrations ${\hat{\text{X}}}_{a}$ of *free* ternary complexes. Therefore, we derive implicit equations to compute these concentrations from measured *total* tRNA concentrations. The details of this derivation are given in the Supporting Information. As a result, we obtain the free concentration ${\hat{\text{X}}}_{b}$ of ternary complex species *b* as an implicit function of all free concentrations ${\hat{\text{X}}}_{a}$ of ternary complex species *a* with *a* = 1, …, 43, the concentration 𝓔^fr^ of free EF-Tu molecules as determined by Eq (S49) in the Supporting Information, the concentration 𝓡 of ribosomes, the codon-dependent probabilities 𝓟_*c*,co_ and 𝓟_*c*,nr_ of cognate and near-cognate accommodation defined in Eqs (15) and (16), and the codon usages *p*_*c*_

$$\begin{array}{c} {\hat{\text{X}}}_{b}={\text{X}}_{b}\;{\left(1+\frac{\omega^{\text{dis}}}{\kappa^{\text{ass}}{𝓔}^{\text{fr}}}+ℛ\left(\Phi_{\text{co}}\sum_{c\in C_{\text{co}}\left(b\right)}\frac{{𝓟}_{c,\text{co}}p_{c}}{\sum_{a\in A_{\text{co}}\left(c\right)}{\hat{\text{X}}}_{a}}+\Phi_{\text{nr}}\sum_{c\in C_{\text{nr}}\;\left(b\right)}\frac{{𝓟}_{c,\text{nr}}p_{c}}{\sum_{a\in A_{\text{nr}}\;\left(c\right)}{\hat{\text{X}}}_{a}}+\omega_{\text{elo}}\tau_{\text{no}}\sum_{c\in C_{\text{no}}\left(b\right)}\frac{{𝓟}_{c,\text{co}}p_{c}}{\sum_{a\in A_{\text{co}}\left(c\right)}{\hat{\text{X}}}_{a}}\right)\right)}^{-1}, \end{array}$$ (28)

where *κ* ^ass^ and *ω* ^dis^ are the binding rate constant and the dissociation rate, respectively, which govern ternary complex formation from free Ef-Tu molecules and aa-tRNAs. For the 2-3-2 pathway of tRNA release from the ribosomal E site, the dimensionless constants Φ_co_ and Φ_nr_ assume the values

$$\Phi_{\text{co}}=\Phi_{\text{co}}^{2-3-2}\equiv 2+\;\omega_{\text{elo}}\;\left(\tau_{\text{co}}+\frac{1}{\omega^{\text{re}}}+\frac{1}{\kappa^{\text{ass}}{𝓔}^{\text{fr}}}\right),$$ (29)

$$\Phi_{\text{nr}}=\Phi_{\text{nr}}^{2-3-2}\equiv 2+\;\omega_{\text{elo}}\;\left(\tau_{\text{nr}}+\frac{1}{\omega^{\text{re}}}+\frac{1}{\kappa^{\text{ass}}{𝓔}^{\text{fr}}}\right),$$ (30)

where ω^re^ is the rate governing the recharging of de-aminoacylated tRNAs by synthetases with new amino acids, see Fig 2. For the 2-1-2 pathway we obtain

$$\Phi_{\text{co}}=\Phi_{\text{co}}^{2-1-2}\equiv 1+\;\omega_{\text{elo}}\;\left(\tau_{\text{co}}+\frac{1}{\omega_{\text{pro}}}+\frac{1}{\omega^{\text{re}}}+\frac{1}{\kappa^{\text{ass}}{𝓔}^{\text{fr}}}\right),$$ (31)

$$\Phi_{\text{nr}}=\Phi_{\text{nr}}^{2-1-2}\equiv 1+\;\omega_{\text{elo}}\;\left(\tau_{\text{nr}}+\frac{1}{\omega_{\text{pro}}}+\frac{1}{\omega^{\text{re}}}+\frac{1}{\kappa^{\text{ass}}{𝓔}^{\text{fr}}}\right),$$ (32)

with constant time scales

$$\tau_{\text{co}}=\;\frac{1}{\omega_{\text{rec}}\pi_{23}\pi_{45}}+\frac{1}{\omega_{23}\pi_{45}}+\frac{1}{\omega_{\text{con}}\pi_{45}}+\frac{1}{\omega_{45}},$$ (33)

$$\tau_{\text{nr}}=\;\frac{1}{\omega_{\text{rec}}\pi_{78}\pi_{9,10}}+\frac{1}{\omega_{78}\pi_{9,10}}+\frac{1}{\omega_{\text{con}}\pi_{9,10}}+\frac{1}{\omega_{9,10}},$$ (34)

$$\tau_{\text{no}}=\;\frac{1}{\omega_{\text{rec}}\pi_{23}\pi_{45}}+\frac{1}{\omega_{\text{off}}\pi_{45}},$$ (35)

where *π*_*ij*_ represents the probability of transition from state (*c*|*i*) to state (*c*|*j*), see definition (Eq 14). Since *E. coli* contains 43 different elongator tRNA species *b*, there are 43 different Eq (28).

**Fig 6:**
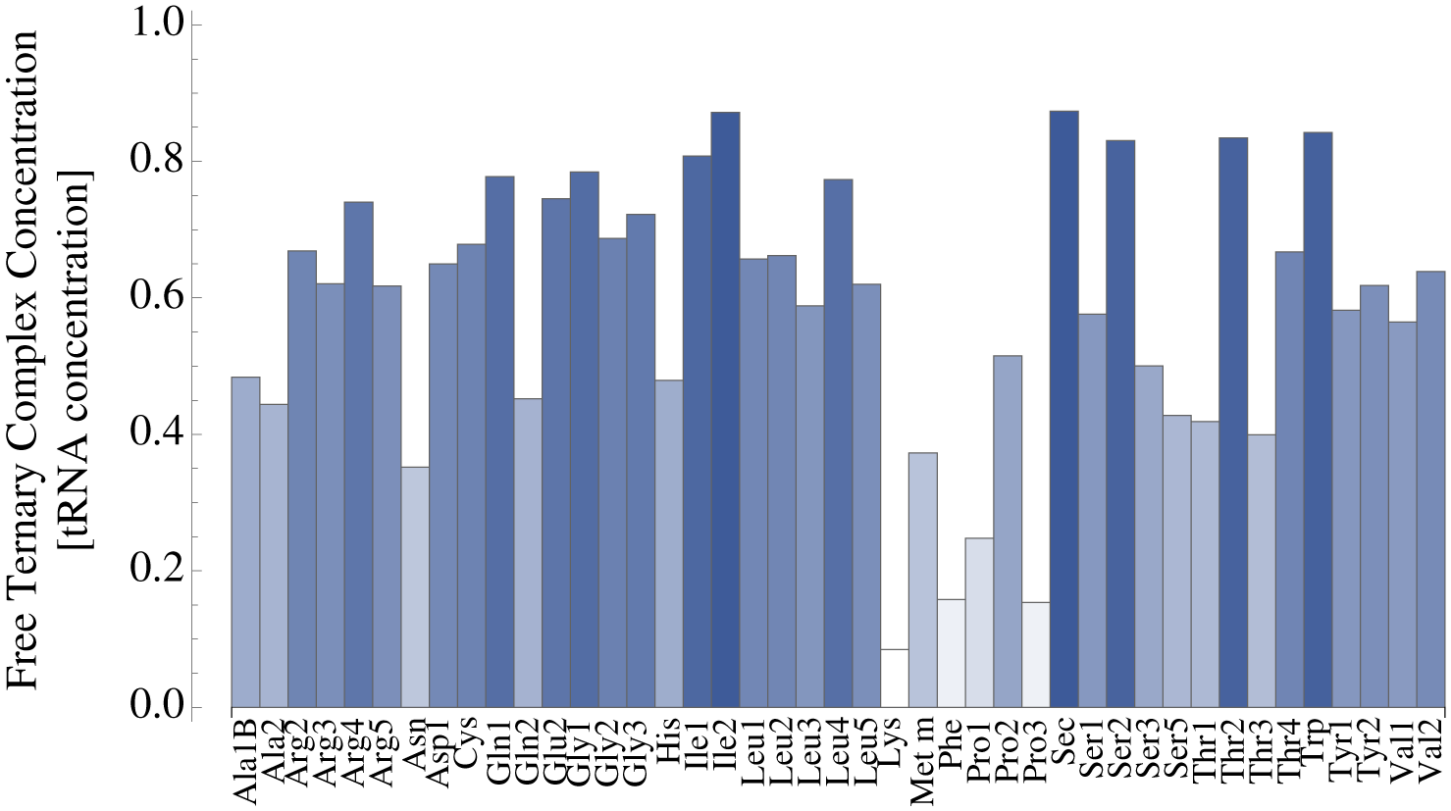
Concentrations of free ternary complexes relative to the total concentrations of the corresponding tRNAs. for all 43 tRNA species in *E. coli* cells growing at a specific rate of 2.5 h^−1^ under the assumption of the 2-3-2 pathway of E-site tRNA release. As an example, about 87% of all tRNA^Ile2^ molecules are part of free ternary complexes. In contrast, only about 8% of all tRNA^Lys^ molecules are contained within free ternary complexes.

For a specific growth rate of 2.5 h^−1^ and the 2-3-2 pathway of E-site tRNA release, Fig 6 shows the resulting concentrations ${\hat{\text{X}}}_{b}$ of free ternary complexes relative to the measured total concentrations X_*b*_ of the corresponding tRNA molecules given in S4 Table in the Supporting Information. We find that the relative concentrations of free ternary complexes strongly depend on the tRNA species: About 87% of all tRNA^Ile2^ molecules are part of free ternary complexes. In contrast, the concentration of free ternary complexes containing tRNA^Lys^ is only about 8% of the total concentration of tRNA^Lys^ molecules. Therefore, the difference between tRNA concentration and free ternary complex concentration is highly significant and cannot be neglected. Concentrations of free ternary complexes for different specific growth rates and both E-site tRNA release mechanisms can be found in S5 and S6 Tables in the Supporting Information.

### Influence of Codon Usage on Dynamics of Translation

In this section, we apply the theory developed in the previous sections to study the influence of codon usage on codon-specific elongation rates. The strong expression of artificially induced genes leads to a shift of the codon usages towards the frequencies that the codons have in the induced gene. Because the codon usages determine how many of the corresponding tRNAs and ternary complexes are bound to ribosomes, they also affect the concentrations of available ternary complexes and ultimately the codon-specific elongation rates as well as missense error frequencies. Therefore, overexpressing a gene can notably change the codon-specific elongation rates with potential consequences for protein folding or ribosome stalling.

**Fig 7:**
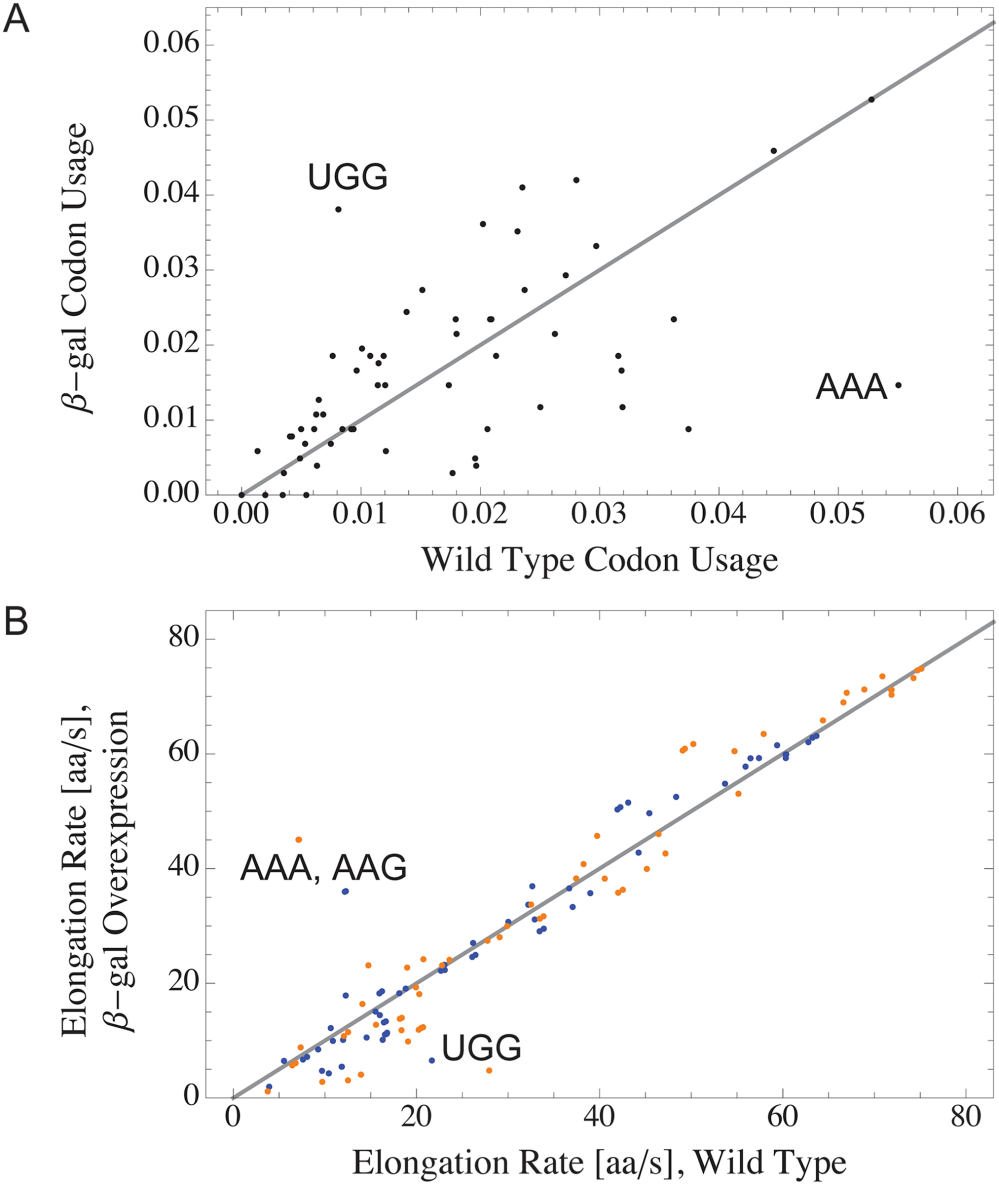
Influence of *β*-galactosidase overexpression on codon-specific elongation rates. (A) Codon usages of all sense codons in wild type *E. coli* cells growing at a specific rate of 2.5 h^−1^ compared to codon usages in *E. coli* cells overexpressing *β*-galactosidase. The largest changes are obtained for the codon usages of AAA and UGG. (B) Elongation rates of all 61 sense codons in wild type *E. coli* cells growing at a specific rate of 2.5 h^−1^ compared to codon-specific elongation rates in *E. coli* cells overexpressing *β*-galactosidase encoded by the lacZ gene, calculated for the 2-1-2 pathway (blue) and the 2-3-2 pathway (orange) of E-site tRNA release. Note that the elongation rates of the codons AAA and AAG are almost identical for both the 2-1-2 pathway (blue) and the 2-3-2 pathway (orange). Except for the codon usages, all parameters used to calculate the elongation rates were identical in all four calculations; in particular, all calculations used the same set of total concentrations of all tRNA species.

To test this hypothesis, we calculated the codon-specific elongation rates using codon usages that are equal to the frequencies of the codons in the *β*-galactosidase gene *lacZ*. This corresponds for example to a cell in which the vast majority of ribosomes are translating *lacZ* mRNAs, i.e., a cell in the limit of very strong *β*-galactosidase overexpression. A direct comparison of the *β*-galactosidase codon frequencies with codon usages from wild type *E. coli* cells growing at a specific growth rate of 2.5 h^−1^ is given in Fig 7(A): the *lacZ* codon usages of codons AAA and UGG differ the most from their corresponding wild type values. The wild type *rare* codon UGG is *abundant* in *lacZ*, which leads to a decrease of its elongation rate in cells overexpressing *β*-galactosidase by about a factor of two when E-site tRNA is released via the 2-1-2 pathway, see Fig 7(B). This codon usage related reduction of elongation rate is even more pronounced if one assumes a 2-3-2 pathway of E-site tRNA release: here, the elongation rate of UGG is decreased by about a factor of six when shifting from wild type to *lacZ* codon usage.

In contrast, the codon AAA is highly abundant in wild type cells but is not a frequent codon in lacZ. Therefore, the AAA elongation rate is increased in *β*-galactosidase overexpressing cells by factors of about two and six for the 2-1-2 and the 2-3-2 pathway of E-site tRNA release, respectively. In addition, the elongation rate of AAG is also increased in *β*-galactosidase overexpressing cells, although its codon usage is comparable to its wild type value. Both AAG and AAA are cognate to tRNA^Lys^ and therefore an increase in free Lys-tRNA^Lys^ ternary complex also increases the elongation rate of AAG.

**Fig 8:**
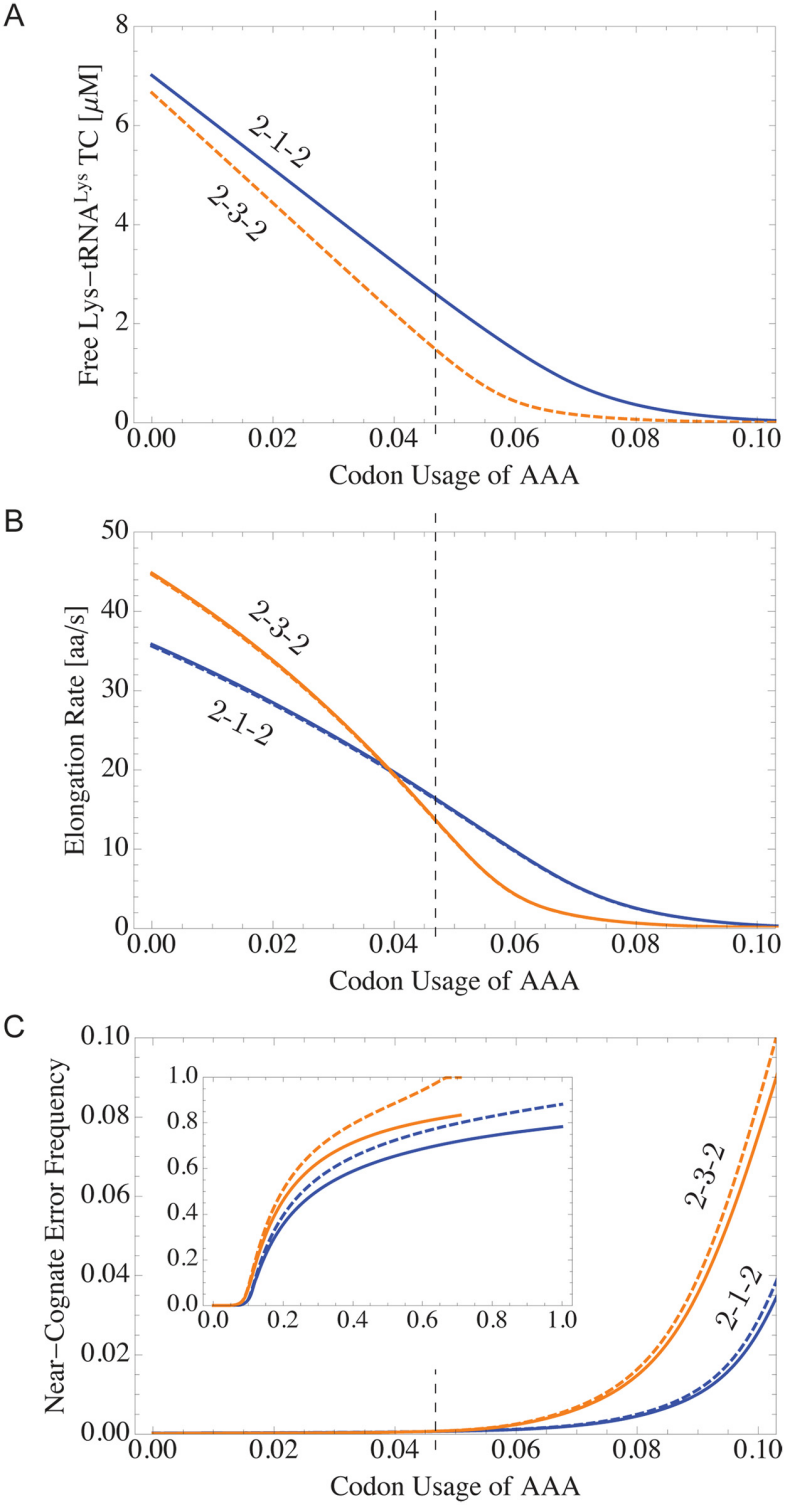
Changing the codon usage of AAA for fixed ratios of the codon usages for all other codons. (A) The concentration of free Lys-tRNA^Lys^ ternary complexes decreases when the codon usage of one of its cognate codons, AAA, increases. (B) Elongation rates of codons AAA (solid line) and AAG (dashed line), both of which are cognate to tRNA^Lys^. The solid and dashed lines coincide almost perfectly. (C) Near-cognate missense error frequencies for both codons (AAA: solid; AAG: dashed). Results are shown for both the 2-1-2 pathway (blue) and the 2-3-2 pathway (orange) of E-site tRNA release. The vertical dashed lines (black) indicate the wild type value 0.0467 of AAA codon usage, see S7 Table in the Supporting Information.

To elucidate the interdependence of codon usage, ternary complex concentration, elongation rate and missense error frequency, we varied the codon usage of one particular codon while keeping the ratios of the codon usages of all other codons at their wild type values. All codon usages were rescaled to fulfill the normalization condition Eq (17). In particular, we have calculated the functional dependence of (i) the concentration of available Lys-tRNA^Lys^ ternary complex, (ii) the codon-specific elongation rates of the corresponding cognate codons AAA and AAG, and (iii) the missense error frequencies of both codons as a function of AAA codon usage for the two alternative pathways of E-site tRNA release, see Fig 8. The concentration of free Lys-tRNA^Lys^ ternary complexes decreases when the codon usage of its cognate codon AAA increases. This depletion of free Lys-tRNA^Lys^ ternary complexes leads to decreasing elongation rates of the codons AAA and AAG. As an additional consequence of the decrease in free Lys-tRNA^Lys^ ternary complex concentration, the near-cognate missense error frequencies of both codons AAA and AAG rise when the codon usage of AAA increases because of the increasing probability that a near-cognate ternary complex gets accommodated in the ribosomal A site.

Our results show that the overexpression of a gene can strongly influence translation dynamics and fidelity due to changes in the codon usages. Altering the codon sequence of the desired gene by using synonymous codons that are apparently faster or less error-prone can in fact lead to slower or more erroneous translation if the resulting codon usages are much higher than in the wild type cell. Thus, instead of replacing all codons coding for a specific amino acid by the same apparently optimal synonymous codon, our results suggest that codon optimization may be improved by the possible substitution of a given codon by several synonymous codons that are cognate to different tRNAs.

## Discussion

It is of great relevance for medical therapies and for the biotechnological production of protein-based substances to understand what determines the speed and accuracy of protein synthesis. To address this long-lasting puzzle, we developed a comprehensive theoretical framework on protein synthesis by ribosomes in bacteria based on current biochemical knowledge about ribosomal kinetics *in vitro* [7, 41–46]. We described translation elongation as a Markov process with 12 different ribosomal transition rates and considered two alternative pathways of tRNA release from the ribosomal E site. The ribosomal transition rates were determined by minimizing the kinetic distance to the measured *in-vitro* rates, a method that was recently introduced in [36]. A fundamental ingredient of our modeling is the dependence of codon-specific elongation rates and missense error frequencies on the concentrations of ternary complexes available for uptake by translating ribosomes. Thus, in contrast to earlier work, we distinguished the total concentrations of tRNA molecules from the concentrations of free ternary complexes and took the binding of aa-tRNAs and tRNAs to active ribosomes as well as the recharging of deacylated tRNAs with new amino acids into account. We analyzed the steady state of the translation process and found that for some tRNA species, e.g. tRNA^Lys^, only a minor fraction of the tRNA molecules are actually incorporated into free ternary complexes and, thus, available for ribosomal uptake. Therefore, calculations of the speed of protein synthesis that are based on total tRNA concentrations instead of free ternary complex concentrations involve systematic errors.

Furthermore, we investigated how elongation rates and missense error frequencies are affected by codon usages. We showed that the overexpression of a single gene leads to altered codon-specific elongation rates such that codons that are slow in wild type cells can become fast in overexpressing cells and *vice versa*. This behavior is predicted to occur when the codon usages in the overexpressed gene are different from those in wild type cells.

The codon-specific Markov process introduced here can be used to study the dependence of the speed and accuracy of *in-vivo* and *in-vitro* protein synthesis on a variety of parameters which have not been considered in this work. For example, one may study how these quantities vary with changes in the overall ternary complex composition or how changes in internal transition rates, arising for example from ribosomal protein or rRNA mutagenesis, affect the synthesis rate.

In the study presented here, we chose to focus on translation in *E. coli* because of the extensive data base available for these cells. However, our theory is quite general and should in principle be applicable to all prokaryotic and eukaryotic cells.

## Supporting Information

S1 Text Supporting Information Text. (PDF)

S1 Table *In-vivo* rates of ribosomal transitions. The values of the overall elongation rate *ω*_elo_ for the four specific growth rates 0.7, 1.07, 1.6, and 2.5 h^−1^ were obtained from the data in [53]. These growth rates have been chosen because the total tRNA concentrations have been measured for these conditions [37] as well. The *in-vivo* rates of ribosomal transitions (with relative standard deviations RSD) were obtained under the assumption of a 2-1-2 (top) or a 2-3-2 (bottom) pathway of tRNA release from the ribosomal E site by minimizing the kinetic distance of *in-vitro* and *in-vivo* rates as described in [36].
(PDF)

S2 Table Codon-specific elongation rates *ω*_*c*,elo_ for all sense codons *c* in *E. coli*, assuming a 2-1-2 pathway of tRNA release from the E site. (PDF)

S3 Table Codon-specific elongation rates *ω*_*c*,elo_ for all sense codons *c* in *E. coli*, assuming a 2-3-2 pathway of tRNA release from the E site. (PDF)

S4 Table *In-vivo* concentrations of all tRNAs, actively translating ribosomes 𝓡, and EF-Tu molecules 𝓔 in *E. coli* for four different specific growth rates. Concentrations of tRNAs from Table 5 in [37], with tRNA^Val2A^ and tRNA^Val2B^ added (Val2), and individual concentrations of tRNA^Gly1^ and tRNA^Gly2^ as well as tRNA^Ile1^ and tRNA^Ile2^ obtained by using corresponding ratios given in [56]. The corresponding *in-vivo* concentrations 𝓡 of active ribosomes are calculated from Table 3 in [37] by taking into account that only 85% of all ribosomes in the cell are active [53]. Furthermore, the total *in-vivo* concentration of EF-Tu can be estimated by interpolating the measured ratios of EF-Tu and ribosome concentrations for different specific growth rates, see [57], and multiplying these ratios by the ribosome concentration 𝓡.
(PDF)

S5 Table Concentrations of free ternary complexes in *E. coli* for four different specific growth rates, assuming a 2-1-2 pathway of tRNA release from the E site. (PDF)

S6 Table Concentrations of free ternary complexes in *E. coli* for four different specific growth rates, assuming a 2-3-2 pathway of tRNA release from the E site. (PDF)

S7 Table *In-vivo* codon usages *p*_*c*_ in percent for all sense codons *c* in *E. coli*. For the specific growth rate of 2.5 h^−1^, the codon usages were determined from relative mRNA abundances of 4215 different genes [58]. In all other cases, data from [37] were used and rescaled to exclude stop codons.
(PDF)
