# Supplementary material for: Protein Synthesis in E. coli: Dependence of Codon-Specific Elongation on tRNA Concentration and Codon Usage
Source: PLoS One. 2015 Aug 13;10(8):e0134994. doi: 10.1371/journal.pone.0134994 (PMC4535986; doi:10.1371/journal.pone.0134994)
Supplement: S1 Text — (PDF) [file pone.0134994.s001.pdf]

Supporting Information: Text

Protein Synthesis in E. coli: Dependence of Codon-specific Elongation on tRNA Concentration and Codon Usage

Sophia Rudorf and Reinhard Lipowsky*

Theory and Bio-Systems, Max Planck Institute of Colloids and Interfaces, Potsdam, Germany

∗ **E-mail: Reinhard.Lipowsky@mpikg.mpg.de**

Codon-Specific Dwell Times

In this section, we derive explicit equations for the dwell times $\mathrm{t}_{(c|i)}$ defined in eq. (8) in the main text. The dwell times $\mathrm{t}_{(c|i)}$ can be calculated from the steady state of the auxiliary Markov process introduced in the main text. In the steady state, the left hand side of eq. (9) in the main text vanishes

$$\frac{\mathrm{d}}{\mathrm{dt}}\,\tilde{\mathrm{P}}_{c,i}(t) = \sum_{j}\left(\tilde{\mathrm{P}}_{c,j}(t)\,\omega_{\mathrm{ji}} - \tilde{\mathrm{P}}_{c,i}(t)\,\omega_{\mathrm{ij}}\right) = 0\,. \tag{S1}$$

This set of equations can be solved for $\tilde{\mathrm{P}}^{\mathrm{st}}_{c,0}$, $\tilde{\mathrm{P}}^{\mathrm{st}}_{c,1}$,..., $\tilde{\mathrm{P}}^{\mathrm{st}}_{c,11}$, where $\tilde{\mathrm{P}}^{\mathrm{st}}_{c,i}$ denotes the steady state value $\tilde{\mathrm{P}}^{\mathrm{st}}_{c,i} \equiv \tilde{\mathrm{P}}_{c,i}(t=\infty)$, and the normalization condition $\sum_i \tilde{\mathrm{P}}_{c,i}(t) = 1$ has to be fulfilled. The steady state probabilities $\tilde{\mathrm{P}}^{\mathrm{st}}_{c,i}$ of the auxiliary process are identical to the fractions of time that the original process spends on average in the states $(c|i)$ with initial state $(c|0)$ and absorbing state $(c'|0')$ which implies the relation (11) in the main text

$$\tilde{\mathrm{P}}^{\mathrm{st}}_{c,i} = \frac{\mathrm{t}_{(c|i)}}{\mathrm{t}_{c,\mathrm{elo}}}$$

with the codon-specific elongation time $\mathrm{t}_{c,\mathrm{elo}}$ defined in eq. (8) in the main text. In other words, the dwell times $\mathrm{t}_{(c|i)}$ follow directly from the solutions of the steady state eqs. (S1).

The resulting dwell time $\mathrm{t}_{(c|0)}$ that the ribosome spends in the free state $(c|0)$ during one complete elongation cycle at codon c is given by

$$\mathrm{t}_{(c|0)} = \frac{1}{\omega_{c,\mathrm{co}} + \omega_{c,\mathrm{nr}}} \tag{S2}$$

with the effective cognate and near-cognate accommodation rates $\omega_{c,\text{co}}$ and $\omega_{c,\text{nr}}$ defined in eq. (12) and eq. (13) in the main text. The dwell time $\mathrm{t}_{(c|11)}$ with a bound non-cognate ternary complex is

$$\mathrm{t}_{(c|11)} = \mathrm{t}_{(c|0)} \frac{\omega_{0,11}}{\omega_{\text{off}}} , \tag{S3}$$

where the dissociation rate ω_{off} is defined in eq. (7) in the main text.

Likewise, the dwell times $\mathrm{t}_{(c|1)}$ to $\mathrm{t}_{(c|5)}$ for the states of the cognate branch can be expressed as

$$\mathrm{t}_{(c|1)} = \mathcal{P}_{c,\text{co}} \frac{1}{\omega_{\text{rec}}} \frac{1}{\pi_{23}} \frac{1}{\pi_{45}} , \tag{S4}$$

$$\mathrm{t}_{(c|2)} = \mathcal{P}_{c,\text{co}} \frac{1}{\omega_{23}} \frac{1}{\pi_{45}} , \tag{S5}$$

$$\mathrm{t}_{(c|3)} = \mathcal{P}_{c,\text{co}} \frac{1}{\omega_{\text{con}}} \frac{1}{\pi_{45}} , \tag{S6}$$

$$\mathrm{t}_{(c|4)} = \mathcal{P}_{c,\text{co}} \frac{1}{\omega_{45}} , \tag{S7}$$

and

$$\mathrm{t}_{(c|5)} = \mathcal{P}_{c,\text{co}} \frac{1}{\omega_{\text{pro}}} , \tag{S8}$$

with the probability $\mathcal{P}_{c,\text{co}}$ for cognate accommodation defined in eq. (15), and ω_{rec}, ω_{con}, and ω_{pro} as in eqs. (4) to (6) in the main text. Finally, the dwell times spent in the near-cognate states $(c|6)$ to $(c|10)$ are given by

$$\mathrm{t}_{(c|6)} = \mathcal{P}_{c,\text{nr}} \frac{1}{\omega_{\text{rec}}} \frac{1}{\pi_{78}} \frac{1}{\pi_{9,10}} , \tag{S9}$$

$$\mathrm{t}_{(c|7)} = \mathcal{P}_{c,\text{nr}} \frac{1}{\omega_{78}} \frac{1}{\pi_{9,10}} , \tag{S10}$$

$$\mathrm{t}_{(c|8)} = \mathcal{P}_{c,\text{nr}} \frac{1}{\omega_{\text{con}}} \frac{1}{\pi_{9,10}} , \tag{S11}$$

$$\mathrm{t}_{(c|9)} = \mathcal{P}_{c,\text{nr}} \frac{1}{\omega_{9,10}} , \tag{S12}$$

and

$$\mathrm{t}_{(c|10)} = \mathcal{P}_{c,\mathrm{nr}} \frac{1}{\omega_{\mathrm{pro}}}, \tag{S13}$$

with the near-cognate accommodation probability $\mathcal{P}_{c,\mathrm{nr}}$ defined in eq. (16) in the main text.

Time Dependent Concentrations of Ribosomal and tRNA Subpopulations

We define the ribosomal and tRNA subpopulations as follows. First, we consider translation in the cell or *in vitro* with fixed time-independent values of the total concentration $\mathcal{R}$ of actively translating ribosomes, of the total concentrations X_a of all tRNA species a, and of the total concentration of all EF-Tu molecules $\mathcal{E}$. Second, the total ribosome concentration $\mathcal{R}$ can be decomposed as

$$\mathcal{R} = \sum_{c=1}^{61} \mathcal{R}_c(t), \tag{S14}$$

where $\mathcal{R}_c(t)$ is the concentration of all ribosomes with codon c in their A sites at time t. The concentration $\mathcal{R}_c(t)$ can be expressed by the marginal probability $\mathrm{P}_c(t)$ to find a ribosome with codon c in its A site

$$\mathcal{R}_c(t) = \mathrm{P}_c(t)\, \mathcal{R}. \tag{S15}$$

Similarly, tRNA molecules belonging to species a can be divided into several subpopulations. The time dependence of all of these tRNA subpopulations is intimately connected to the ribosomal dynamics described by the Markov process as defined in the main text. We denote the concentrations of free ternary complexes by $\hat{\mathrm{X}}_a$ and of cognate, near-cognate, and non-cognate ternary complexes after initial binding to ribosomes by $\hat{\mathrm{X}}_a^{(1)}$, $\hat{\mathrm{X}}_a^{(6)}$, and $\hat{\mathrm{X}}_a^{(11)}$, respectively. Accordingly, the concentrations of cognate ternary complexes that are bound to ribosomes in state $(c|2)$, and of near-cognate ternary complexes bound to ribosomes in state $(c|7)$ are denoted by $\hat{\mathrm{X}}_a^{(2)}$ and $\hat{\mathrm{X}}_a^{(7)}$, respectively. The time-dependent concentrations

of the initially bound ternary complexes then fulfill

$$\frac{\mathrm{d}}{\mathrm{dt}}\,\hat{\mathrm{X}}_a^{(1)}(t) = \kappa_{\mathrm{on}}\,\hat{\mathrm{X}}_a(t) \sum_{c\in\mathbf{C}_{\mathrm{co}}(a)} \mathrm{P}_{0|c}(t)\,\mathcal{R}_c(t) + \hat{\mathrm{X}}_a^{(2)}(t)\,\omega_{21} - \hat{\mathrm{X}}_a^{(1)}(t)\,(\omega_{\mathrm{off}} + \omega_{\mathrm{rec}})\ , \tag{S16}$$

$$\frac{\mathrm{d}}{\mathrm{dt}}\,\hat{\mathrm{X}}_a^{(6)}(t) = \kappa_{\mathrm{on}}\,\hat{\mathrm{X}}_a(t) \sum_{c\in\mathbf{C}_{\mathrm{nr}}(a)} \mathrm{P}_{0|c}(t)\,\mathcal{R}_c(t) + \hat{\mathrm{X}}_a^{(7)}(t)\,\omega_{76} - \hat{\mathrm{X}}_a^{(6)}(t)\,(\omega_{\mathrm{off}} + \omega_{\mathrm{rec}})\ , \tag{S17}$$

$$\frac{\mathrm{d}}{\mathrm{dt}}\,\hat{\mathrm{X}}_a^{(11)}(t) = \kappa_{\mathrm{on}}\,\hat{\mathrm{X}}_a(t) \sum_{c\in\mathbf{C}_{\mathrm{no}}(a)} \mathrm{P}_{0|c}(t)\,\mathcal{R}_c(t) - \hat{\mathrm{X}}_a^{(11)}(t)\,\omega_{\mathrm{off}}\ , \tag{S18}$$

with transition rates ω_{ij} as defined in Fig. 4 (A) in the main text and the conditional probability $\mathrm{P}_{0|c}(t)$ that a ribosome dwelling on codon c attains the free state $(c|0)$ with empty A site. The concentration $\mathcal{R}_c(t)$ of all ribosomes with codon c in their A sites is defined by eq. (S15).

After entering the cognate branch of the codon-specific Markov process shown in Fig. 4 (A) in the main text, the ribosome visits the states $(c|2)$ to $(c|4)$. The dynamics of the concentrations of the associated ternary complex subpopulations follow

$$\frac{\mathrm{d}}{\mathrm{dt}}\,\hat{\mathrm{X}}_a^{(2)}(t) = \hat{\mathrm{X}}_a^{(1)}(t)\,\omega_{\mathrm{rec}} - \hat{\mathrm{X}}_a^{(2)}(t)\,(\omega_{21} + \omega_{23})\ , \tag{S19}$$

$$\frac{\mathrm{d}}{\mathrm{dt}}\,\hat{\mathrm{X}}_a^{(3)}(t) = \hat{\mathrm{X}}_a^{(2)}(t)\,\omega_{23} - \hat{\mathrm{X}}_a^{(3)}(t)\,\omega_{\mathrm{con}}\ , \tag{S20}$$

$$\frac{\mathrm{d}}{\mathrm{dt}}\,\hat{\mathrm{X}}_a^{(4)}(t) = \hat{\mathrm{X}}_a^{(3)}(t)\,\omega_{\mathrm{con}} - \hat{\mathrm{X}}_a^{(4)}(t)\,(\omega_{40} + \omega_{45})\ . \tag{S21}$$

Similarly, ribosomes in the near-cognate branch attain states $(c|7)$ to $(c|9)$ and the concentrations of the ternary complex subpopulations bound to these ribosomes are denoted by $\hat{\mathrm{X}}_a^{(7)}$, $\hat{\mathrm{X}}_a^{(8)}$, and $\hat{\mathrm{X}}_a^{(9)}$, respectively. Their time dependence is determined by

$$\frac{\mathrm{d}}{\mathrm{dt}}\,\hat{\mathrm{X}}_a^{(7)}(t) = \hat{\mathrm{X}}_a^{(6)}(t)\,\omega_{\mathrm{rec}} - \hat{\mathrm{X}}_a^{(7)}(t)\,(\omega_{76} + \omega_{78})\ , \tag{S22}$$

$$\frac{\mathrm{d}}{\mathrm{dt}}\,\hat{\mathrm{X}}_a^{(8)}(t) = \hat{\mathrm{X}}_a^{(7)}(t)\,\omega_{78} - \hat{\mathrm{X}}_a^{(8)}(t)\,\omega_{\mathrm{con}}\ , \tag{S23}$$

$$\frac{\mathrm{d}}{\mathrm{dt}}\,\hat{\mathrm{X}}_a^{(9)}(t) = \hat{\mathrm{X}}_a^{(8)}(t)\,\omega_{\mathrm{con}} - \hat{\mathrm{X}}_a^{(9)}(t)\,(\omega_{90} + \omega_{9,10})\ . \tag{S24}$$

Furthermore, concentrations of aa-tRNAs of species a that got fully accommodated in ribosomal A sites are denoted by $\mathrm{X}_a^{\mathrm{A}}$, of tRNAs bound to ribosomal P sites by $\mathrm{X}_a^{\mathrm{P}}$, and of tRNAs bound to ribosomal E

sites by $\mathrm{X}_a^{\mathrm{E}}$. Their dynamics are described by

$$\frac{\mathrm{d}}{\mathrm{dt}}\,\mathrm{X}_a^{\mathrm{A}}(t) = \hat{\mathrm{X}}_a^{(4)}(t)\,\omega_{45} + \hat{\mathrm{X}}_a^{(9)}(t)\,\omega_{9,10} - \mathrm{X}_a^{\mathrm{A}}(t)\,\omega_{\mathrm{pro}}\,, \tag{S25}$$

$$\frac{\mathrm{d}}{\mathrm{dt}}\,\mathrm{X}_a^{\mathrm{P}}(t) = \mathrm{X}_a^{\mathrm{A}}(t)\,\omega_{\mathrm{pro}} - \mathrm{X}_a^{\mathrm{P}}(t)\,\omega_{\mathrm{elo}}\,, \tag{S26}$$

$$\frac{\mathrm{d}}{\mathrm{dt}}\,\mathrm{X}_a^{\mathrm{E}}(t) = \mathrm{X}_a^{\mathrm{P}}(t)\,\omega_{\mathrm{elo}} - \mathrm{X}_a^{\mathrm{E}}(t)\,\frac{\omega_{\mathrm{pro}}\omega_{\mathrm{elo}}}{\omega_{\mathrm{pro}} - \omega_{\mathrm{elo}}}\,. \tag{S27}$$

As shown in Fig. 2 in the main text, de-aminoacylated tRNAs released from ribosomes are recharged by synthetases with new amino acids with rate ω^{re}. The concentration of tRNAs being in this process of recharging is called $\mathrm{X}_a^{\mathrm{re}}$

$$\frac{\mathrm{d}}{\mathrm{dt}}\,\mathrm{X}_a^{\mathrm{re}}(t) = \mathrm{X}_a^{\mathrm{E}}(t)\,\frac{\omega_{\mathrm{pro}}\omega_{\mathrm{elo}}}{\omega_{\mathrm{pro}} - \omega_{\mathrm{elo}}} - \mathrm{X}_a^{\mathrm{re}}(t)\,\omega^{\mathrm{re}}\,. \tag{S28}$$

Note that eqs. (S27) and (S28) are given for the 2-3-2 pathway of E-site tRNA release, for which the E-site tRNA does not leave the ribosome until a new aa-tRNA has been accommodated in the ribosomal A site. In contrast, when the 2-1-2 pathway is considered, tRNAs are released rapidly from the E site so that always $\mathrm{X}_a^{\mathrm{E}}(t) \simeq 0$ and the first term on the right hand side of eq. (S28) has to be replaced by the last term of eq. (S26).

After being recharged, an aa-tRNA needs to bind an EF-Tu molecule to form a new ternary complex, see upper right of Fig. 2. The concentration $\mathrm{X}_a^{\mathrm{ch}}$ refers to free aa-tRNAs that are not bound to EF-Tu molecules. Furthermore, we denote by $\mathcal{E}^{\mathrm{fr}}$ the concentration of free EF-Tu molecules. Free EF-Tus bind to aa-tRNAs with binding rate constant κ^{ass} to form ternary complexes that in turn decay with rate ω^{dis} which leads to

$$\frac{\mathrm{d}}{\mathrm{dt}}\,\mathrm{X}_a^{\mathrm{ch}}(t) = \mathrm{X}_a^{\mathrm{re}}(t)\,\omega^{\mathrm{re}} + \hat{\mathrm{X}}_a(t)\,\omega^{\mathrm{dis}} - \kappa^{\mathrm{ass}}\mathcal{E}^{\mathrm{fr}}(t)\mathrm{X}_a^{\mathrm{ch}}(t)\,, \tag{S29}$$

$$\frac{\mathrm{d}}{\mathrm{dt}}\,\mathcal{E}^{\mathrm{fr}}(t) = \hat{\mathrm{X}}_a^{(4)}(t)\,\omega_{45} + \hat{\mathrm{X}}_a^{(9)}(t)\,\omega_{9,10} + \hat{\mathrm{X}}_a(t)\,\omega^{\mathrm{dis}} - \kappa^{\mathrm{ass}}\mathcal{E}^{\mathrm{fr}}(t)\mathrm{X}_a^{\mathrm{ch}}(t)\,, \tag{S30}$$

where the first two terms in the second equation describe the ternary complex disintegration and EF-Tu release from the ribosome, two processes that occur as soon as the aa-tRNA gets fully accommodated in the ribosomal A site, see Fig. 1 in the main text.

Finally, because the number of molecules of each tRNA species a is taken to be conserved, the tRNA

subpopulations add up to the total concentration X_a which implies the decomposition

$$\begin{aligned}\mathrm{X}_a =&\hat{\mathrm{X}}_a(t) + \sum_{i=1}^{4} \hat{\mathrm{X}}_a^{(i)}(t) + \sum_{i=6}^{9} \hat{\mathrm{X}}_a^{(i)}(t) + \hat{\mathrm{X}}_a^{(11)}(t) \\ &+ \mathrm{X}_a^{\mathrm{A}}(t) + \mathrm{X}_a^{\mathrm{P}}(t) + \mathrm{X}_a^{\mathrm{E}}(t) + \mathrm{X}_a^{\mathrm{re}}(t) + \mathrm{X}_a^{\mathrm{ch}}(t)\,. \end{aligned} \tag{S31}$$

Steady State Concentrations of tRNA Subpopulations

If we assume that translation is in a steady state, the solution of the Markov process for cell-wide translation leads to expressions for the different tRNA subpopulations in terms of the concentrations of all free ternary complexes. In a steady state, the left hand sides of eqs. (S16) to (S30) vanish, and we can use eqs. (S2), (S15) and (27) in the main text to obtain

$$\mathrm{P}_{0|c}(t)\,\mathcal{R}_c(t) = \mathrm{P}_{c,0}\,\mathcal{R} = \frac{\omega_{\mathrm{elo}}\,p_c}{\omega_{c,\mathrm{co}} + \omega_{c,\mathrm{nr}}}\mathcal{R} \tag{S32}$$

where the effective cognate and near-cognate accommodation rates $\omega_{c,\mathrm{co}}$ and $\omega_{c,\mathrm{nr}}$ are defined in eqs. (15) and (16) in the main text, respectively. In steady state, expression (S32) replaces the corresponding terms in eqs. (S16) to (S18).

We focus on one particular tRNA species b. The steady state solutions for cognate bound ternary complexes of species b can be expressed by the free concentrations $\hat{\mathrm{X}}_b$ and $\hat{\mathrm{X}}_a$ of ternary complex species b and all other ternary complex species a, respectively,

$$\hat{\mathrm{X}}_b^{(1)} = \frac{\omega_{\mathrm{elo}}}{\omega_{\mathrm{rec}}\pi_{23}\pi_{45}} \sum_{c\in\mathbf{C}_{\mathrm{co}}(b)} \frac{\mathcal{P}_{c,\mathrm{co}}\,p_c}{\sum\limits_{a\in\mathbf{A}_{\mathrm{co}}(c)} \hat{\mathrm{X}}_a}\,\mathcal{R}\,\hat{\mathrm{X}}_b\,, \tag{S33}$$

$$\hat{\mathrm{X}}_b^{(2)} = \frac{\omega_{\mathrm{elo}}}{\omega_{23}\pi_{45}} \sum_{c\in\mathbf{C}_{\mathrm{co}}(b)} \frac{\mathcal{P}_{c,\mathrm{co}}\,p_c}{\sum\limits_{a\in\mathbf{A}_{\mathrm{co}}(c)} \hat{\mathrm{X}}_a}\,\mathcal{R}\,\hat{\mathrm{X}}_b\,, \tag{S34}$$

$$\hat{\mathrm{X}}_b^{(3)} = \frac{\omega_{\mathrm{elo}}}{\omega_{\mathrm{con}}\pi_{45}} \sum_{c\in\mathbf{C}_{\mathrm{co}}(b)} \frac{\mathcal{P}_{c,\mathrm{co}}\,p_c}{\sum\limits_{a\in\mathbf{A}_{\mathrm{co}}(c)} \hat{\mathrm{X}}_a}\,\mathcal{R}\,\hat{\mathrm{X}}_b\,, \tag{S35}$$

$$\hat{\mathrm{X}}_b^{(4)} = \frac{\omega_{\mathrm{elo}}}{\omega_{45}} \sum_{c\in\mathbf{C}_{\mathrm{co}}(b)} \frac{\mathcal{P}_{c,\mathrm{co}}\,p_c}{\sum\limits_{a\in\mathbf{A}_{\mathrm{co}}(c)} \hat{\mathrm{X}}_a}\,\mathcal{R}\,\hat{\mathrm{X}}_b\,, \tag{S36}$$

where the probability of cognate accommodation $\mathcal{P}_{c,\mathrm{co}}$ is defined in eq. (15) in the main text. Similarly, tRNAs of species b that are contained in near-cognate bound ternary complexes yield

$$\hat{\mathrm{X}}_b^{(6)} = \frac{\omega_{\mathrm{elo}}}{\omega_{\mathrm{rec}}\pi_{78}\pi_{9,10}} \sum_{c\in\mathbf{C}_{\mathrm{nr}}(b)} \frac{\mathcal{P}_{c,\mathrm{nr}}\, p_c}{\sum\limits_{a\in\mathbf{A}_{\mathrm{nr}}(c)} \hat{\mathrm{X}}_a} \mathcal{R}\,\hat{\mathrm{X}}_b\,, \tag{S37}$$

$$\hat{\mathrm{X}}_b^{(7)} = \frac{\omega_{\mathrm{elo}}}{\omega_{78}\pi_{9,10}} \sum_{c\in\mathbf{C}_{\mathrm{nr}}(b)} \frac{\mathcal{P}_{c,\mathrm{nr}}\, p_c}{\sum\limits_{a\in\mathbf{A}_{\mathrm{nr}}(c)} \hat{\mathrm{X}}_a} \mathcal{R}\,\hat{\mathrm{X}}_b\,, \tag{S38}$$

$$\hat{\mathrm{X}}_b^{(8)} = \frac{\omega_{\mathrm{elo}}}{\omega_{\mathrm{con}}\pi_{9,10}} \sum_{c\in\mathbf{C}_{\mathrm{nr}}(b)} \frac{\mathcal{P}_{c,\mathrm{nr}}\, p_c}{\sum\limits_{a\in\mathbf{A}_{\mathrm{nr}}(c)} \hat{\mathrm{X}}_a} \mathcal{R}\,\hat{\mathrm{X}}_b\,, \tag{S39}$$

and

$$\hat{\mathrm{X}}_b^{(9)} = \frac{\omega_{\mathrm{elo}}}{\omega_{9,10}} \sum_{c\in\mathbf{C}_{\mathrm{nr}}(b)} \frac{\mathcal{P}_{c,\mathrm{nr}}\, p_c}{\sum\limits_{a\in\mathbf{A}_{\mathrm{nr}}(c)} \hat{\mathrm{X}}_a} \mathcal{R}\,\hat{\mathrm{X}}_b\,. \tag{S40}$$

For non-cognate bound ternary complexes of species b the following expression holds

$$\hat{\mathrm{X}}_b^{(11)} = \omega_{\mathrm{elo}} \left(\frac{1}{\omega_{\mathrm{rec}}\pi_{23}\pi_{45}} + \frac{1}{\omega_{\mathrm{off}}\pi_{45}} \right) \sum_{c\in\mathbf{C}_{\mathrm{no}}(b)} \frac{\mathcal{P}_{c,\mathrm{co}}\, p_c}{\sum\limits_{a\in\mathbf{A}_{\mathrm{co}}(c)} \hat{\mathrm{X}}_a} \mathcal{R}\,\hat{\mathrm{X}}_b\,. \tag{S41}$$

The steady state concentration $\mathrm{X}_b^{\mathrm{P}}$ of tRNAs of species b accommodated in ribosomal P sites is given by

$$\mathrm{X}_b^{\mathrm{P}} = \left(\sum_{c\in\mathbf{C}_{\mathrm{co}}(b)} \frac{\mathcal{P}_{c,\mathrm{co}}\, p_c}{\sum\limits_{a\in\mathbf{A}_{\mathrm{co}}(c)} \hat{\mathrm{X}}_a} + \sum_{c\in\mathbf{C}_{\mathrm{nr}}(b)} \frac{\mathcal{P}_{c,\mathrm{nr}}\, p_c}{\sum\limits_{a\in\mathbf{A}_{\mathrm{nr}}(c)} \hat{\mathrm{X}}_a} \right) \mathcal{R}\,\hat{\mathrm{X}}_b\,, \tag{S42}$$

which is related to the steady state concentrations $\mathrm{X}_b^{\mathrm{A}}$, $\mathrm{X}_b^{\mathrm{E}}$, $\mathrm{X}_b^{\mathrm{re}}$, and $\mathrm{X}_b^{\mathrm{ch}}$ of tRNAs of species b in ribosomal A and E sites as well as in de-aminoacylated and aminoacylated form

$$\mathrm{X}_b^{\mathrm{A}} = \frac{\omega_{\mathrm{elo}}}{\omega_{\mathrm{pro}}} \mathrm{X}_b^{\mathrm{P}}\,, \tag{S43}$$

$$\mathrm{X}_b^{\mathrm{E}} = \left(1 - \frac{\omega_{\mathrm{elo}}}{\omega_{\mathrm{pro}}} \right) \mathrm{X}_b^{\mathrm{P}}\,, \tag{S44}$$

$$\mathrm{X}_b^{\mathrm{re}} = \frac{\omega_{\mathrm{elo}}}{\omega^{\mathrm{re}}} \mathrm{X}_b^{\mathrm{P}}\,, \tag{S45}$$

$$\mathrm{X}_b^{\mathrm{ch}} = \frac{\omega^{\mathrm{dis}}\,\hat{\mathrm{X}}_b}{\kappa^{\mathrm{ass}}\,\mathcal{E}^{\mathrm{fr}}} + \frac{\omega_{\mathrm{elo}}\,\mathrm{X}_b^{\mathrm{P}}}{\kappa^{\mathrm{ass}}\,\mathcal{E}^{\mathrm{fr}}}\,. \tag{S46}$$

Note that eq. (S44) only holds for the 2-3-2 pathway of E-site tRNA release, as in case of the 2-1-2 pathway the concentration of E site bound tRNA $\mathrm{X}_b^{\mathrm{E}}$ is always zero.

Derivation of the Steady State Concentrations of Free Ternary Complexes

In the previous section, the steady state solutions of the concentrations $\hat{\mathrm{X}}_b^{(1)}, \ldots \mathrm{X}_b^{\mathrm{ch}}$ of the various subpopulations of tRNA species b are presented in terms of the concentrations $\hat{\mathrm{X}}_a$ and $\hat{\mathrm{X}}_b$ of free ternary complexes, see eqs. (S33) to (S46). In this section, we will derive an implicit expression for the free concentration $\hat{\mathrm{X}}_b$ of ternary complex species b in steady state.

The total steady state concentration X^{ch} of all charged aa-tRNAs depends on the steady state concentration $\mathcal{E}^{\mathrm{fr}}$ of free EF-Tu molecules, the total concentration X^{P} of P site accommodated tRNAs, and the total concentration $\hat{\mathrm{X}} = \sum_a \hat{\mathrm{X}}_a$ of free ternary complexes

$$\mathrm{X}^{\mathrm{ch}} = \sum_a \mathrm{X}_a^{\mathrm{ch}} = \frac{\omega^{\mathrm{dis}}\,\hat{\mathrm{X}}}{\kappa^{\mathrm{ass}}\,\mathcal{E}^{\mathrm{fr}}} + \frac{\omega_{\mathrm{elo}}\,\mathrm{X}^{\mathrm{P}}}{\kappa^{\mathrm{ass}}\,\mathcal{E}^{\mathrm{fr}}}\,, \tag{S47}$$

where $\mathrm{X}^{\mathrm{P}} = \sum_a \mathrm{X}_a^{\mathrm{P}}$ equals the total concentration $\mathcal{R}$ of ribosomes as the P site of every active ribosome is occupied by a tRNA. Using eq. (S47), the concentration $\mathcal{E}^{\mathrm{fr}}$ of free EF-Tu molecules can be deduced from the assumption that the total concentration $\mathcal{E}$ of EF-Tu molecules is conserved, i.e., that the sum of all concentrations of ternary complexes and $\mathcal{E}^{\mathrm{fr}}$ must equal the total concentration $\mathcal{E}$ of EF-Tu

$$\begin{aligned}\mathcal{E}^{\mathrm{fr}} &= \mathcal{E} - \left(\hat{\mathrm{X}} + \sum_{i=1}^{4}\hat{\mathrm{X}}^{(i)} + \sum_{i=6}^{9}\hat{\mathrm{X}}^{(i)} + \hat{\mathrm{X}}^{(11)}\right)\\ &= \mathcal{E} - \left(\mathrm{X} - \mathrm{X}^{\mathrm{A}} - \mathrm{X}^{\mathrm{P}} - \mathrm{X}^{\mathrm{E}} - \mathrm{X}^{\mathrm{re}} - \mathrm{X}^{\mathrm{ch}}\right)\,,\end{aligned} \tag{S48}$$

where $\mathrm{X} = \sum_a \mathrm{X}_a$ is the sum of all total tRNA concentrations defined in eq. (S31). The steady state total concentration $\mathrm{X}^{\mathrm{A}} + \mathrm{X}^{\mathrm{E}} + \mathrm{X}^{\mathrm{re}} = \sum_a (\mathrm{X}_a^{\mathrm{A}} + \mathrm{X}_a^{\mathrm{E}} + \mathrm{X}_a^{\mathrm{re}})$ of A- and E-site tRNAs and tRNAs before recharging is equal to $\left(\frac{\omega_{\mathrm{elo}}}{\omega_{\mathrm{pro}}} + \frac{\omega_{\mathrm{elo}}}{\omega^{\mathrm{re}}}\right)\mathcal{R}$ and $\left(1 + \frac{\omega_{\mathrm{elo}}}{\omega^{\mathrm{re}}}\right)\mathcal{R}$ for the 2-1-2 and the 2-3-2 pathway, respectively, see eqs. (S43) to (S45) in the previous section. Thus, the concentration of free EF-Tu molecules can be written as

$$\mathcal{E}^{\mathrm{fr}} = \frac{\beta}{2} + \sqrt{\frac{\beta^2}{4} + \frac{\omega^{\mathrm{dis}}}{\kappa^{\mathrm{ass}}}\,\hat{\mathrm{X}} + \frac{\omega_{\mathrm{elo}}}{\kappa^{\mathrm{ass}}}\,\mathcal{R}}\,, \tag{S49}$$

with

$$\beta \equiv \begin{cases} \mathcal{E} - \mathrm{X} + \left(1 + \frac{\omega_{\mathrm{elo}}}{\omega_{\mathrm{pro}}} + \frac{\omega_{\mathrm{elo}}}{\omega^{\mathrm{re}}}\right)\mathcal{R} & \text{for the 2-1-2 pathway,} \\ \mathcal{E} - \mathrm{X} + \left(2 + \frac{\omega_{\mathrm{elo}}}{\omega^{\mathrm{re}}}\right)\mathcal{R} & \text{for the 2-3-2 pathway.} \end{cases} \tag{S50}$$

Finally, the free concentration $\hat{\mathrm{X}}_b$ of ternary complex species b can be deduced from the steady state concentrations of all tRNA subpopulations given in eqs. (S33) to (S46) together with constraint (S31) that the total number of molecules of tRNA species b is conserved. The result is presented in eq. (28) in the main text.

Parameters for tRNA Recharging and Ternary Complex Formation

To our knowledge, the binding of de-aminoacylated tRNAs to synthetases and the rate ω^{re} of their subsequent recharging with amino acids has not been measured *in vivo* for each kind of tRNAs and each of the twenty different synthetases. Therefore, we estimated $1/\omega^{\mathrm{re}} = 10\,\mathrm{ms}$ to be the same for all tRNA species as in [1, 2].

The disassembly of ternary complexes into EF-Tu and aa-tRNA is characterized by the dissociation rate ω^{dis}. Likewise, ternary complex assembly from EF-Tu and aa-tRNA is governed by the association rate constant κ^{ass} and the concentration $\mathcal{E}^{\mathrm{fr}}$ of free EF-Tu molecules. We use the values $\kappa^{\mathrm{ass}} = 1\mu\mathrm{M}^{-1}\mathrm{s}^{-1}$ and $\omega^{\mathrm{dis}} = 0.01\mathrm{s}^{-1}$ as in [3] and assume that these rates also hold for *in-vivo* translation. The free EF-Tu concentration depends on the specific growth rate and is calculated from solving eq. (S48). The results

are $\mathcal{E}^{\rm fr} = 5.4\,\mu$M for 0.7 h^{-1}, 14$\,\mu$M for 1.07 h^{-1}, 20$\,\mu$M for 1.6 h^{-1}, and 23$\,\mu$M for 2.5 h^{-1}.

References

1. Cestari I, Stuart K (2013) A spectrophotometric assay for quantitative measurement of aminoacyl-tRNA synthetase activity. Journal of Biomolecular Screening 18: 490-497.

2. Cvetesic N, Perona JJ, Gruic-Sovulj I (2012) Kinetic partitioning between synthetic and editing pathways in class I aminoacyl-tRNA synthetases occurs at both pre-transfer and post-transfer hydrolytic steps. Journal of Biological Chemistry 287: 25381–25394.

3. Mittelstaet J, Konevega AL, Rodnina MV (2013) A kinetic safety gate controlling the delivery of unnatural amino acids to the ribosome. Journal of the American Chemical Society 135: 17031 - 17038.
