# Supplementary material for: Protein Synthesis in E. coli: Dependence of Codon-Specific Elongation on tRNA Concentration and Codon Usage
Source: PLoS One. 2015 Aug 13;10(8):e0134994. doi: 10.1371/journal.pone.0134994 (PMC4535986; doi:10.1371/journal.pone.0134994)
Supplement: S1 Table — The values of the overall elongation rate ω elo for the four specific growth rates 0.7, 1.07, 1.6, and 2.5 h−1 were obtained from the data in [53]. These growth rates have been chosen because the total tRNA concentrations have been measured for these conditions [37] as well. The in-vivo rates of ribosomal transitions (with relative standard deviations RSD) were obtained under the assumption of a 2-1-2 (top) or a 2-3-2 (bottom) pathway of tRNA release from the ribosomal E site by minimizing the kinetic distance of in-vitro and in-vivo rates as described in [36]. (PDF) [file pone.0134994.s002.pdf]

Supporting Information: S1 Table

Protein Synthesis in E. coli: Dependence of Codon-specific Elongation on tRNA Concentration and Codon Usage

Sophia Rudorf and Reinhard Lipowsky*

Theory and Bio-Systems, Max Planck Institute of Colloids and Interfaces, Potsdam, Germany

∗ **E-mail: Reinhard.Lipowsky@mpikg.mpg.de**

Table S1. *In-vivo* rates of ribosomal transitions. The values of the overall elongation rate ω_{elo} for the four specific growth rates 0.7, 1.07, 1.6, and 2.5 h^{-1} were obtained from the data in [1]. These growth rates have been chosen because the total tRNA concentrations have been measured for these conditions [2] as well. The *in-vivo* rates of ribosomal transitions (with relative standard deviations RSD) were obtained under the assumption of a 2-1-2 (top) or a 2-3-2 (bottom) pathway of tRNA release from the ribosomal E site by minimizing the kinetic distance of *in-vitro* and *in-vivo* rates as described in [3].

E site release	Rates	Specific growth rate [h^{-1}]				RSD	Units
		0.7	1.07	1.6	2.5		
	ω_{elo}	15	18	22	22		aa s^{-1}
	κ_{on}	94	94	94	94	0.1	$\mu M^{-1} s^{-1}$
2-1-2	ω_{off}	2000	2100	2900	3400	0.4	s^{-1}
	ω_{rec}	3000	3100	4200	4900	0.3	s^{-1}
	ω_{21}	2	2	2	2	0.3	s^{-1}
	ω_{23}	1600	1600	1700	1800	0.3	s^{-1}
	ω_{con}	510	520	560	570		s^{-1}
	ω_{45}	300	320	390	400	0.2	s^{-1}
	ω_{40}	1	1	1	1		s^{-1}
	ω_{76}	3900	4000	5500	6500	0.3	s^{-1}
	ω_{78}	4	4	4	4	0.3	s^{-1}
	$\omega_{9,10}$	0.27	0.27	0.27	0.27	0.2	s^{-1}
	ω_{90}	6	6	7	7	0.2	s^{-1}
	ω_{pro}	200	220	250	260	0.5	s^{-1}
2-3-2	ω_{off}	2100	2200	3300	4500	0.4	s^{-1}
	ω_{rec}	3200	3400	4900	7000	0.3	s^{-1}
	ω_{21}	2	2	2	2	0.3	s^{-1}
	ω_{23}	1600	1700	1800	1800	0.3	s^{-1}
	ω_{con}	510	530	570	600		s^{-1}
	ω_{45}	310	330	410	450	0.2	s^{-1}
	ω_{40}	1	1	1	1		s^{-1}
	ω_{76}	4100	4300	6300	8900	0.3	s^{-1}
	ω_{78}	4	4	4	4	0.3	s^{-1}
	$\omega_{9,10}$	0.27	0.27	0.27	0.27	0.2	s^{-1}
	ω_{90}	6	7	7	8	0.2	s^{-1}
	ω_{pro}	210	220	260	280	0.5	s^{-1}

References

1. Liang ST, Xu YC, Dennis PP, Bremer H (2000) mRNA composition and control of bacterial gene expression. Journal of Bacteriology 182: 3037-3044.

2. Dong H, Nilsson L, Kurland CG (1996) Co-variation of tRNA abundance and codon usage in Escherichia coli at different growth rates. Journal of Molecular Biology 260: 649-663.

3. Rudorf S, Thommen M, Rodnina MV, Lipowsky R (2014) Deducing the kinetics of protein synthesis *in vivo* from the transition rates measured *in vitro*. PLoS Computational Biology 10: e1003909.
