# Supplementary material for: Protein Synthesis in E. coli: Dependence of Codon-Specific Elongation on tRNA Concentration and Codon Usage
Source: PLoS One. 2015 Aug 13;10(8):e0134994. doi: 10.1371/journal.pone.0134994 (PMC4535986; doi:10.1371/journal.pone.0134994)
Supplement: S2 Table — (PDF) [file pone.0134994.s003.pdf]

Supporting Information: S2 Table

Protein Synthesis in E. coli: Dependence of Codon-specific Elongation on tRNA Concentration and Codon Usage

Sophia Rudorf and Reinhard Lipowsky*

Theory and Bio-Systems, Max Planck Institute of Colloids and Interfaces, Potsdam, Germany

∗ **E-mail: Reinhard.Lipowsky@mpikg.mpg.de**

Table S2. Codon-specific elongation rates $\omega_{c,\mathrm{elo}}$ for all sense codons c in *E. coli*, assuming a 2-1-2 pathway of tRNA release from the E site.
All rates in $[\mathrm{s}^{-1}]$.

	Specific growth rate $[\mathrm{h}^{-1}]$					Specific growth rate $[\mathrm{h}^{-1}]$			
	0.7	1.07	1.6	2.5		0.7	1.07	1.6	2.5
AAA	15.3	16.4	14.6	12.3	GAA	38.9	40.1	53.8	63.7
AAC	7.9	10.2	14.8	16.6	GAC	20.9	21.1	30.0	39.0
AAG	15.3	16.3	14.5	12.2	GAG	38.6	39.9	53.3	63.2
AAU	7.8	10.1	14.6	16.4	GAU	19.6	19.9	28.3	37.1
ACA	11.8	11.5	17.7	22.7	GCA	26.7	32.5	37.5	43.1
ACC	10.6	11.1	12.3	16.2	GCC	5.1	6.4	9.9	10.4
ACG	19.6	19.2	27.4	32.9	GCG	25.9	31.5	36.6	42.3
ACU	19.2	19.4	25.6	32.2	GCU	25.7	31.3	36.6	41.9
AGA	12.2	12.8	16.2	15.9	GGA	17.1	17.4	24.9	23.1
AGC	15.8	15.7	19.2	16.7	GGC	36.8	39.1	44.2	55.9
AGG	8.7	8.3	13.0	10.6	GGG	26.9	27.5	38.2	36.7
AGU	15.6	15.5	19.0	16.6	GGU	35.0	37.3	42.1	53.7
AUA	3.0	3.1	4.7	5.5	GUA	31.7	28.0	45.4	48.4
AUC	31.9	34.3	46.8	59.4	GUC	14.8	16.0	20.8	23.1
AUG	5.7	7.0	10.2	10.9	GUG	29.2	25.6	42.2	45.4
AUU	30.5	33.1	44.9	57.4	GUU	38.6	36.4	53.5	56.5
CAA	10.9	14.2	12.4	16.0	UAA		*stop*		
CAC	4.0	6.3	7.6	11.9	UAC	22.5	22.2	33.5	33.9
CAG	6.4	8.2	13.0	16.3	UAG		*stop*		
CAU	4.0	6.3	7.6	11.8	UAU	22.0	21.8	33.0	33.4
CCA	3.9	5.6	4.7	3.9	UCA	22.0	20.7	26.6	26.2
CCC	11.3	9.8	17.7	14.5	UCC	9.0	9.6	11.9	12.3
CCG	9.8	15.3	10.7	9.7	UCG	24.3	23.3	29.8	30.0
CCU	13.7	13.8	20.2	16.8	UCU	26.4	25.8	32.5	32.6
CGA	40.9	41.9	58.1	62.8	UGA		*stop*		
CGC	38.9	39.9	56.0	60.4	UGC	19.6	19.8	27.1	26.4
CGG	12.3	6.6	10.7	9.3	UGG	11.5	13.6	17.2	21.7
CGU	38.8	39.8	55.9	60.3	UGU	19.2	19.5	26.7	26.1
CUA	9.1	9.3	11.6	12.0	UUA	15.5	17.3	16.5	15.5
CUC	13.1	14.7	17.7	18.8	UUC	7.4	10.7	9.8	8.1
CUG	43.7	46.5	56.6	60.3	UUG	34.1	36.1	44.1	44.3
CUU	12.6	14.2	16.9	18.1	UUU	6.9	10.1	9.2	7.6
