# Supplementary material for: Protein Synthesis in E. coli: Dependence of Codon-Specific Elongation on tRNA Concentration and Codon Usage
Source: PLoS One. 2015 Aug 13;10(8):e0134994. doi: 10.1371/journal.pone.0134994 (PMC4535986; doi:10.1371/journal.pone.0134994)
Supplement: S3 Table — (PDF) [file pone.0134994.s004.pdf]

Supporting Information: S3 Table

Protein Synthesis in E. coli: Dependence of Codon-specific Elongation on tRNA Concentration and Codon Usage

Sophia Rudorf and Reinhard Lipowsky*

Theory and Bio-Systems, Max Planck Institute of Colloids and Interfaces, Potsdam, Germany

∗ **E-mail: Reinhard.Lipowsky@mpikg.mpg.de**

Table S3. Codon-specific elongation rates $\omega_{c,\mathrm{elo}}$ for all sense codons c in *E. coli*, assuming a 2-3-2 pathway of tRNA release from the E site.
All rates in [s^{-1}].

	Specific growth rate [h^{-1}]					Specific growth rate [h^{-1}]			
	0.7	1.07	1.6	2.5		0.7	1.07	1.6	2.5
AAA	15.2	15.4	12.0	7.2	GAA	40.0	41.9	58.1	75.1
AAC	7.6	9.6	14.8	18.4	GAC	21.3	21.4	31.9	47.2
AAG	15.1	15.4	11.9	7.1	GAG	39.7	41.7	57.6	74.7
AAU	7.5	9.5	14.6	18.2	GAU	20.0	20.2	30.2	45.2
ACA	12.2	12.0	19.3	27.8	GCA	27.1	33.2	39.2	50.2
ACC	10.6	10.8	12.1	19.0	GCC	5.1	6.4	10.6	12.5
ACG	20.3	20.4	30.3	40.6	GCG	26.3	32.2	38.2	49.3
ACU	19.6	19.6	26.7	38.2	GCU	26.1	32.1	38.4	49.1
AGA	12.9	14.1	18.8	20.8	GGA	18.0	19.2	28.6	29.9
AGC	16.4	16.8	21.6	20.7	GGC	37.7	40.7	47.2	66.6
AGG	9.2	9.2	15.2	14.1	GGG	28.2	30.0	43.2	46.4
AGU	16.3	16.7	21.4	20.5	GGU	36.0	38.9	45.2	64.4
AUA	3.1	3.4	5.5	7.4	GUA	32.6	28.8	48.9	57.9
AUC	32.8	35.8	50.6	70.9	GUC	15.3	16.9	23.1	29.1
AUG	5.7	6.8	10.4	12.5	GUG	30.0	26.3	45.6	54.7
AUU	31.3	34.5	48.6	68.9	GUU	39.6	37.7	57.7	67.0
CAA	11.3	15.4	13.9	20.3	UAA		*stop*		
CAC	3.8	6.0	7.3	13.9	UAC	23.2	23.5	37.1	42.5
CAG	6.3	7.8	13.3	19.1	UAG		*stop*		
CAU	3.8	5.9	7.3	13.9	UAU	22.7	23.0	36.5	42.0
CCA	3.8	5.5	4.3	3.8	UCA	22.8	22.1	29.6	32.5
CCC	11.8	10.6	20.2	18.4	UCC	9.3	9.9	12.7	14.7
CCG	9.8	15.4	10.0	9.7	UCG	25.3	24.9	33.2	37.4
CCU	14.1	14.4	22.2	20.2	UCU	27.4	27.3	35.6	39.7
CGA	42.0	43.7	62.7	74.3	UGA		*stop*		
CGC	40.0	41.8	60.6	71.9	UGC	20.5	21.6	30.8	33.9
CGG	13.0	7.3	12.5	12.1	UGG	11.9	14.5	19.4	27.9
CGU	39.9	41.6	60.5	71.8	UGU	20.1	21.2	30.3	33.5
CUA	9.5	10.0	12.9	15.6	UUA	16.2	18.8	18.8	19.9
CUC	13.7	15.9	20.0	23.6	UUC	7.2	10.4	9.1	6.8
CUG	45.0	48.8	61.2	71.9	UUG	35.5	38.9	49.3	55.2
CUU	13.1	15.4	19.2	22.8	UUU	6.7	9.8	8.5	6.4
