# Supplementary material for: Protein Synthesis in E. coli: Dependence of Codon-Specific Elongation on tRNA Concentration and Codon Usage
Source: PLoS One. 2015 Aug 13;10(8):e0134994. doi: 10.1371/journal.pone.0134994 (PMC4535986; doi:10.1371/journal.pone.0134994)
Supplement: S4 Table — Concentrations of tRNAs from Table 5 in [37], with tRNAVal2A and tRNAVal2B added (Val2), and individual concentrations of tRNAGly1 and tRNAGly2 as well as tRNAIle1 and tRNAIle2 obtained by using corresponding ratios given in [56]. The corresponding in-vivo concentrations 𝓡 of active ribosomes are calculated from Table 3 in [37] by taking into account that only 85% of all ribosomes in the cell are active [53]. Furthermore, the total in-vivo concentration of EF-Tu can be estimated by interpolating the measured ratios of EF-Tu and ribosome concentrations for different specific growth rates, see [57], and multiplying these ratios by the ribosome concentration 𝓡. (PDF) [file pone.0134994.s005.pdf]

Supporting Information: S4 Table

Protein Synthesis in E. coli: Dependence of Codon-specific Elongation on tRNA Concentration and Codon Usage

Sophia Rudorf and Reinhard Lipowsky*

Theory and Bio-Systems, Max Planck Institute of Colloids and Interfaces, Potsdam, Germany

∗ **E-mail: Reinhard.Lipowsky@mpikg.mpg.de**

Table S4. *In-vivo* concentrations of all tRNAs, actively translating ribosomes $\mathcal{R}$, and EF-Tu molecules $\mathcal{E}$ in *E. coli* for four different specific growth rates. Concentrations of tRNAs from Table 5 in [1], with $tRNA^{Val2A}$ and $tRNA^{Val2B}$ added (Val2), and individual concentrations of $tRNA^{Gly1}$ and $tRNA^{Gly2}$ as well as $tRNA^{Ile1}$ and $tRNA^{Ile2}$ obtained by using corresponding ratios given in [2]. The corresponding *in-vivo* concentrations $\mathcal{R}$ of active ribosomes are calculated from Table 3 in [1] by taking into account that only 85% of all ribosomes in the cell are active [3]. Furthermore, the total *in-vivo* concentration of EF-Tu can be estimated by interpolating the measured ratios of EF-Tu and ribosome concentrations for different specific growth rates, see [4], and multiplying these ratios by the ribosome concentration $\mathcal{R}$. All concentrations in [µM].

	Specific growth rate [h^{-1}]			
	0.7	1.07	1.6	2.5
Ala1B	11.73	14.06	17.52	20.97
Ala2	2.12	2.33	3.19	3.57
Arg2	14.54	15.54	23.77	25.57
Arg3	2.61	1.45	2.26	2.3
Arg4	2.35	2.64	3.26	3.52
Arg5	1.57	1.61	2.46	2.2
Asn	3.86	4.35	6.1	7.29
Asp1	8.13	8.42	12.04	15.46
Cys	4.88	5.23	7.04	7.07
Gln1	2.72	3.63	3.17	4.38
Gln2	3.08	3.47	5.07	6.27
Glu2	15.58	16.71	24.12	29.35
Gly1	2.87	3.10	4.38	4.43
Gly2	4.31	4.64	6.57	6.65
Gly3	15.21	16.75	19.84	24.96
His	2.19	2.63	3.35	4.38
Ile1	11.29	12.61	18.02	23.56
Ile2	0.56	0.63	0.90	1.18
Leu1	14.91	16.76	21.32	22.2
Leu2	3.47	4.04	4.72	5.93
Leu3	2.49	2.62	3.19	3.17
Leu4	6.33	6.97	9.66	9.3
Leu5	3.47	4.07	3.65	3.78

	Specific growth rate [h^{-1}]			
	0.7	1.07	1.6	2.5
Lys	6.8	7.35	8.73	10.43
Met m	2.59	2.91	4.10	4.43
Phe	3.6	4.29	4.69	5.11
Pro1	2.44	3.51	2.75	2.67
Pro2	2.51	2.26	4.01	3.75
Pro3	1.89	2.22	2.55	2.56
Sec	0.86	0.96	1.05	1.04
Ser1	5.56	5.47	6.98	7.36
Ser2	1.04	1.17	1.37	1.45
Ser3	4.39	4.53	5.4	5.67
Ser5	2.6	2.87	3.68	4.03
Thr1	0.41	0.54	0.56	0.67
Thr2	2	2.11	2.67	3.12
Thr3	3.73	3.87	4.86	5.54
Thr4	3.17	3.25	4.99	6.89
Trp	2.78	3.35	4.15	5.02
Tyr1	2.41	2.7	4.61	4.19
Tyr2	3.86	3.75	5.22	5.04
Val1	12.07	11.07	18.99	20.39
Val2	4.39	5.02	6.31	7.21
$\mathcal{R}$	18.57	23.57	33.85	44.56
$\mathcal{E}$	152.22	181.57	236.69	257.88

References

1. Dong H, Nilsson L, Kurland CG (1996) Co-variation of tRNA abundance and codon usage in Escherichia coli at different growth rates. Journal of Molecular Biology 260: 649-663.

2. Ikemura T (1981) Correlation between the abundance of Escherichia coli transfer RNAs and the occurrence of the respective codons in its protein genes: A proposal for a synonymous codon choice that is optimal for the E. coli translational system. Journal of Molecular Biology 151: 389 - 409.

3. Liang ST, Xu YC, Dennis PP, Bremer H (2000) mRNA composition and control of bacterial gene expression. Journal of Bacteriology 182: 3037-3044.

4. Neidhardt FC, Bloch PL, Pedersen S, Reeh S (1977) Chemical measurement of steady-state levels of 10 aminoacyl transfer ribonucleic-acid synthetases in Escherichia coli. Journal of Bacteriology 129: 378-387.
