# Supplementary material for: Protein Synthesis in E. coli: Dependence of Codon-Specific Elongation on tRNA Concentration and Codon Usage
Source: PLoS One. 2015 Aug 13;10(8):e0134994. doi: 10.1371/journal.pone.0134994 (PMC4535986; doi:10.1371/journal.pone.0134994)
Supplement: S6 Table — (PDF) [file pone.0134994.s007.pdf]

Supporting Information: S6 Table

Protein Synthesis in E. coli: Dependence of Codon-specific Elongation on tRNA Concentration and Codon Usage

Sophia Rudorf and Reinhard Lipowsky*

Theory and Bio-Systems, Max Planck Institute of Colloids and Interfaces, Potsdam, Germany

∗ E-mail: Reinhard.Lipowsky@mpikg.mpg.de

Table S6. Concentrations of free ternary complexes in *E. coli* for four different specific growth rates, assuming a 2-3-2 pathway of tRNA release from the E site.
All concentrations in [µM].

	Specific growth rate [h^{-1}]					Specific growth rate [h^{-1}]			
	0.7	1.07	1.6	2.5		0.7	1.07	1.6	2.5
Ala1B	5.43	7.14	7.70	8.48	Leu5	2.66	3.20	2.79	2.26
Ala2	0.77	1.00	1.49	1.42	Lys	2.43	2.50	1.65	0.74
Arg2	9.54	10.00	15.36	15.90	Met m	0.96	1.18	1.62	1.53
Arg3	2.27	1.22	1.92	1.40	Phe	1.14	1.74	1.31	0.74
Arg4	2.08	2.33	2.84	2.41	Pro1	1.04	1.87	0.87	0.68
Arg5	1.42	1.44	2.19	1.52	Pro2	1.83	1.65	3.00	2.07
Asn	1.16	1.52	2.13	2.15	Pro3	0.58	0.87	0.58	0.39
Asp1	3.97	4.03	5.74	7.82	Sec	0.79	0.87	0.95	0.92
Cys	3.84	4.12	5.53	4.78	Ser1	3.89	3.79	4.65	4.02
Gln1	1.84	2.64	2.05	2.46	Ser2	0.88	0.99	1.15	1.14
Gln2	0.99	1.28	1.96	2.29	Ser3	3.03	3.15	3.65	2.70
Glu2	9.61	10.19	14.44	17.98	Ser5	1.39	1.51	1.75	1.57
Gly1	2.52	2.70	3.82	3.44	Thr1	0.16	0.21	0.17	0.23
Gly2	3.61	3.90	5.55	4.51	Thr2	1.60	1.69	2.14	2.23
Gly3	9.08	10.06	10.53	14.33	Thr3	1.46	1.47	1.50	1.94
His	0.57	0.93	1.03	1.61	Thr4	1.89	1.89	2.78	3.33
Ile1	6.47	7.38	10.41	14.56	Trp	1.96	2.48	3.00	3.50
Ile2	0.46	0.52	0.74	0.79	Tyr1	1.52	1.70	2.98	2.68
Leu1	10.17	11.51	13.94	14.00	Tyr2	2.44	2.36	3.38	3.23
Leu2	2.42	2.93	3.33	3.12	Val1	7.04	5.92	10.78	10.72
Leu3	1.60	1.72	2.00	1.88	Val2	2.71	3.05	3.84	4.03
Leu4	5.46	5.99	8.21	7.47					
