# Supplementary material for: Protein Synthesis in E. coli: Dependence of Codon-Specific Elongation on tRNA Concentration and Codon Usage
Source: PLoS One. 2015 Aug 13;10(8):e0134994. doi: 10.1371/journal.pone.0134994 (PMC4535986; doi:10.1371/journal.pone.0134994)
Supplement: S7 Table — For the specific growth rate of 2.5 h−1, the codon usages were determined from relative mRNA abundances of 4215 different genes [58]. In all other cases, data from [37] were used and rescaled to exclude stop codons. (PDF) [file pone.0134994.s008.pdf]

Supporting Information: S7 Table

Protein Synthesis in E. coli: Dependence of Codon-specific Elongation on tRNA Concentration and Codon Usage

Sophia Rudorf and Reinhard Lipowsky*

Theory and Bio-Systems, Max Planck Institute of Colloids and Interfaces, Potsdam, Germany

∗ **E-mail: Reinhard.Lipowsky@mpikg.mpg.de**

Table S7. *In-vivo* codon usages p_c in percent for all sense codons c in *E. coli.* For the specific growth rate of 2.5 h^{-1}, the codon usages were determined from relative mRNA abundances of 4215 different genes [1]. In all other cases, data from [2] were used and rescaled to exclude stop codons.

	Specific growth rate [h^{-1}]					Specific growth rate [h^{-1}]			
	0.7	1.07	1.6	2.5		0.7	1.07	1.6	2.5
AAA	4.67	4.94	5.24	5.50	GAA	5.43	5.54	5.72	4.46
AAC	2.84	2.88	2.93	2.72	GAC	2.98	3.11	3.26	2.63
AAG	1.28	1.38	1.50	1.96	GAG	1.69	1.71	1.75	1.74
AAU	0.89	0.78	0.65	0.96	GAU	2.36	2.25	2.13	2.35
ACA	0.33	0.30	0.27	0.42	GCA	2.23	2.25	2.33	2.50
ACC	2.69	2.73	2.77	2.09	GCC	1.86	1.69	1.48	1.51
ACG	0.70	0.62	0.52	1.01	GCG	2.97	2.86	2.75	2.37
ACU	1.52	1.69	1.85	1.97	GCU	3.05	3.26	3.51	3.74
AGA	0.10	0.08	0.07	0.54	GGA	0.25	0.22	0.18	0.63
AGC	1.15	1.08	1.00	1.38	GGC	3.58	3.58	3.58	2.97
AGG	0.01	0.01	0.00	0.34	GGG	0.43	0.36	0.28	0.92
AGU	0.36	0.30	0.24	0.61	GGU	3.94	4.07	4.26	3.62
AUA	0.09	0.08	0.06	0.20	GUA	1.72	1.88	2.01	2.06
AUC	3.80	3.94	4.17	3.18	GUC	1.06	0.98	0.89	1.08
AUG	2.25	2.25	2.25	2.08	GUG	2.05	1.90	1.79	1.79
AUU	2.06	1.94	1.79	1.80	GUU	3.33	3.58	3.85	3.19
CAA	0.97	0.90	0.81	1.14	UAC	1.65	1.68	1.70	1.15
CAC	1.40	1.40	1.42	1.20	UAU	1.00	0.90	0.79	0.65
CAG	2.90	2.85	2.79	2.81	UCA	0.36	0.31	0.26	0.41
CAU	0.88	0.82	0.73	0.76	UCC	1.16	1.22	1.24	1.21
CCA	0.65	0.64	0.61	0.68	UCG	0.54	0.46	0.38	0.50
CCC	0.28	0.21	0.14	0.43	UCU	1.36	1.42	1.50	1.77
CCG	2.94	2.91	2.92	2.31	UGC	0.51	0.48	0.44	0.68
CCU	0.49	0.48	0.47	0.72	UGG	0.93	0.87	0.81	0.81
CGA	0.12	0.10	0.08	0.35	UGU	0.40	0.37	0.33	0.49
CGC	2.25	2.25	2.19	2.02	UUA	0.55	0.47	0.36	0.75
CGG	0.15	0.12	0.09	0.53	UUC	2.27	2.26	2.29	2.13
CGU	3.37	3.68	3.99	3.16	UUG	0.63	0.58	0.50	0.62
CUA	0.19	0.15	0.11	0.13	UUU	1.16	1.04	0.88	1.19
CUC	0.59	0.56	0.51	0.84					
CUG	6.10	6.17	6.21	5.28					
CUU	0.53	0.47	0.40	0.94					

References

1. Zhang G, Fedyunin I, Kirchner S, Xiao C, Valleriani A, et al. (2012) FANSe: an accurate algorithm for quantitative mapping of large scale sequencing reads. Nucleic Acids Research 40.

2. Dong H, Nilsson L, Kurland CG (1996) Co-variation of tRNA abundance and codon usage in Escherichia coli at different growth rates. Journal of Molecular Biology 260: 649-663.
